# Eigengene networks for studying the relationships between co-expression modules

**DOI:** 10.1186/1752-0509-1-54

**Published:** 2007-11-21

**Authors:** Peter Langfelder, Steve Horvath

**Affiliations:** 1 Department of Human Genetics, University of California, Los Angeles, CA 90095, USA; 2 Department of Human Genetics and Department of Biostatistics, University of California, Los Angeles, CA 90095, USA

## Abstract

**Background:**

There is evidence that genes and their protein products are organized into functional modules according to cellular processes and pathways. Gene co-expression networks have been used to describe the relationships between gene transcripts. Ample literature exists on how to detect biologically meaningful modules in networks but there is a need for methods that allow one to study the relationships between modules.

**Results:**

We show that network methods can also be used to describe the relationships between co-expression modules and present the following methodology. First, we describe several methods for detecting modules that are shared by two or more networks (referred to as consensus modules). We represent the gene expression profiles of each module by an eigengene. Second, we propose a method for constructing an eigengene network, where the edges are undirected but maintain information on the sign of the co-expression information. Third, we propose methods for differential eigengene network analysis that allow one to assess the preservation of network properties across different data sets. We illustrate the value of eigengene networks in studying the relationships between consensus modules in human and chimpanzee brains; the relationships between consensus modules in brain, muscle, liver, and adipose mouse tissues; and the relationships between male-female mouse consensus modules and clinical traits. In some applications, we find that module eigengenes can be organized into higher level clusters which we refer to as meta-modules.

**Conclusion:**

Eigengene networks can be effective and biologically meaningful tools for studying the relationships between modules of a gene co-expression network. The proposed methods may reveal a higher order organization of the transcriptome. R software tutorials, the data, and supplementary material can be found at the following webpage: .

## Background

Gene co-expression networks constructed from gene expression microarray data capture the relationships between transcripts [1-7]. From the point of view of individual genes ('from below'), modules are groups of highly interconnected genes that may form a biological pathway. From the point of view of systems biology ('from above'), functional modules bridge the gap between individual genes and emergent global properties [8-10]. Here we view modules as basic system components (*i.e.*, nodes of a network) and describe their relationships using network language. We find that co-expression modules may form a biologically meaningful meta-network that reveals a higher-order organization of the transcriptome. We refer to modules in a meta-network of modules as *meta-modules*.

Our analysis can be viewed as a network reduction scheme that reduces a gene co-expression network involving thousands of genes to an orders of magnitude smaller meta-network involving module representatives (one eigengene per module). We refer to the resulting network as eigengene network. Using eigengene neworks, we will show that the information captured by co-expression modules is far richer than a catalogue of module membership.

As a motivating example, consider the comparison between gene co-expression networks in human and chimpanzee brains. Using gene expression microarray data corresponding to different brain regions, Oldham *et al *[11] found relatively large modules that are preserved between human and chimpanzee brains. Only one human brain module (corresponding to genes expressed in the cortex) was not preserved in chimpanzee brains. The original analysis focused on human modules and assessed their preservation in a corresponding chimpanzee co-expression network. We refer to such an analysis as a standard *marginal module analysis *since it simply determines whether a set of modules can be found in another network. Here we pursue a more comprehensive analysis that not only quantifies module preservation but also determines *inter*modular preservation. We refer to modules that are preserved among data sets as *consensus modules*. In our applications, we show that two consensus modules may be highly related to each other in one data set but unrelated in another. Inter-modular relationships are biologically interesting because changes in pathway dependencies may reflect biological perturbations.

In this work we present methods a) for finding consensus modules across multiple networks, b) for describing the relationship between consensus modules (eigengene networks), and c) for assessing whether the relationship between consensus modules is preserved across different networks (differential eigengene network analysis).

## Results

### Eigengene networks

**Figure 1:**
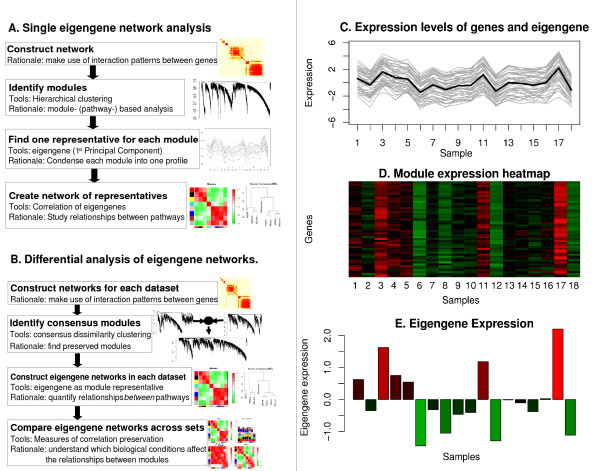
**Overview of eigengene networks**. A. Flowchart of the construction and analysis of an eigengene network based on a single data set. B. Analogous flowchart for constructing and analyzing *consensus *eigengene networks based on multiple data sets. C.–E. Illustrating the notion of eigengene as a representative of an entire gene co-expression module. C. Expression levels (*y*-axis) of module genes (grey lines) and the eigengene (black line) across microarray samples (*x*-axis). The plot shows that an eigengene is highly correlated with the expression profiles of the genes in the module. D. Heatmap of the gene expressions (rows correspond to genes, columns to samples, red denotes over-expression, green under-expression). E. Expression levels (*y*-axis) of the corresponding eigengene across the samples (*x*-axis). Whenever the module gene expression are high (red), the module eigengene are high and similarly for low (green) gene expressions.

Many module detection methods identify groups of genes whose expression profiles are highly correlated. For such modules, one can summarize the module expression profile by one representative gene: the module eigengene. An intuitive explanation of module eigengenes is provided in Figures 1C–E. Specifically, we define the module eigengene as the first right-singular vector of the standardized module expression data (Methods, Eq. 29). Eigengenes of different modules often exhibit correlations which we use to define *eigengene networks*. Figure 1A outlines our approach for constructing an eigengene network corresponding to the modules of a single gene co-expression network. We index the eigengenes by capital letters *I*, *J*,...; for example, *E*_*J *_denotes the (module) eigengene of the *J*-th module. We define the connection strength (adjacency) between eigengenes *I *and *J *as

$$a_{Eigen,IJ}=\frac{1+\text{cor}(E_{I},E_{J})}{2}.$$

**Figure 2:**
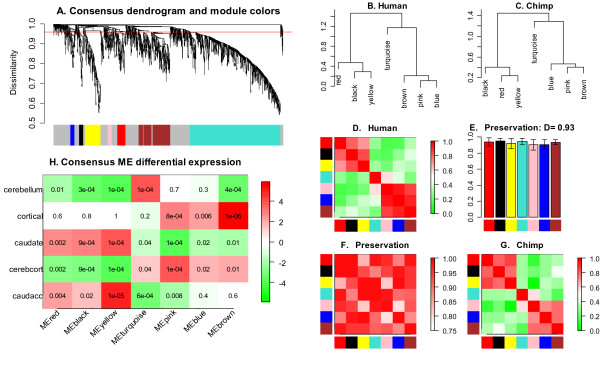
**Differential eigengene network analysis in human and chimp brain samples**. A. Hierarchical clustering dendrogram of genes for identifying consensus modules (see text). Branches of the dendrogram, cut at the red line, correspond to consensus modules. Genes in each module are assigned the same color, shown in the color band below the dendrogram. Genes not assigned to any of the modules are colored grey. B., C. Clustering dendrograms of consensus module eigengenes for identifying meta-modules. The same three meta-modules (major branches) are evident in both dendrograms. D. Heatmap of eigengene adjacencies in the consensus eigengene network in human samples. Each row and column corresponds to one eigengene (labeled by consensus module color). Within the heatmap, red indicates high adjacency (positive correlation) and green low adjacency (negative correlation) as shown by the color legend. G. Corresponding plot for the chimp samples. E. Preservation measure for each consensus eigengene. Each colored bar corresponds to the eigengene of the corresponding color. The height of the bar (*y*-axis) gives the eigengene preservation measure (16). *D *denotes the overall preservation of the eigengene networks, Eq. (17). F. Heatmap of adjacencies in the preservation network *Preserv*^*human*,*chimp*^, Eq. (15). Each row and column corresponds to a consensus module; saturation of the red color encodes adjacency according to the color legend. H. Characterizing consensus modules by differential expression of their corresponding eigengenes in the various brain areas from which samples were taken. Red means over-expression, green under-expression; numbers in each cell give the corresponding *t*-test *p*-value. Each column corresponds to an eigengene and each row corresponds to a brain area. Caudacc, caudate nucleus and anterior cingulate cortex; cerebcort, cerebellum and cortex; caudate, caudate nucleus.

Thus, the eigengene network *A*_*Eigen *_= (*a*_*Eigen*,*IJ*_) is a special case of a signed weighted gene co-expression network (*β *= 1 in Eq. 26, Methods). We use a signed co-expression network because the sign of the correlation between eigengenes carries important biological information in our applications. We use a *weighted *gene co-expression network to describe the relationships between modules since this maintains the continuous nature of the co-expression information. Examples of two different visualization methods of eigengene networks are shown in Fig. 2C,D and 2E,H.

For the *I*-th module eigengene, we define the scaled connectivity (degree) *C*_*I*_(*A*_*Eigen*_) as mean connection strength with the other eigengenes:

$$C_{I}(A_{Eigen})\equiv \frac{1}{2}+\frac{\sum_{J\neq I}\text{cor}(E_{I},E_{J})}{2\left(N-1\right)},$$

where *N *denotes the number of module eigengenes. Note that the scaled connectivity *C*_*I*_(*A*_*Eigen*_) is close to 1 if the *I*-th eigengene has a high positive correlation with most other eigengenes.

The density *D*(*A*_*Eigen*_) of the eigengene network is defined as as the average scaled connectivity (Eq. 9):

$$D(A_{Eigen})\equiv \frac{1}{N}\sum_{I}C_{I}(A_{Eigen})=\frac{1}{2}+\frac{\sum_{I}\sum_{J\neq I}\text{cor}(E_{I},E_{J})}{2N(N-1)}.$$

The density *D*(*A*_*Eigen*_) is close to 1 if most eigengenes have high positive correlations with each other.

#### Meta-modules in a single eigengene network

Since eigengenes form a network, one can use a module detection procedure to identify modules comprised of eigengenes. We refer to modules in an eigengene network as meta-modules. Meta-modules may reveal a higher order organization among gene co-expression modules. We use average linkage hierarchical clustering to define meta-modules as branches of the resulting cluster tree (Methods, Eq. 21). The resulting meta-modules are sets of positively correlated eigengenes.

### Differential eigengene network analysis

Several recent works have described differential network analysis methods for gene co-expression networks [11-13]. Here we propose methods for the differential analysis of eigengene networks. An overview is shown in Figure 1B. We start by defining and detecting consensus modules, *i.e.*, modules that are shared by two or more gene co-expression networks. Consensus modules may represent biological pathways that are shared among the compared data sets. Study of their relationships, represented by consensus eigengene networks, may reveal important differences in pathway regulation under different conditions. Detection of consensus modules proceeds by defining a suitable consensus dissimilarity (Methods, Eq. 22) and using it as input to hierarchical clustering. To compare the consensus eigengene networks (Eq. 1) of two data sets whose adjacency matrices are $A_{Eigen}^{\left(1\right)}$ and $A_{Eigen}^{\left(2\right)}$, we make use of the *preservation network Preserv*^(1,2) ^= *Preserv*($A_{Eigen}^{\left(1\right)}$, $A_{Eigen}^{\left(2\right)}$), in which adjacencies are defined as

$$Preserv_{IJ}^{\left(1,2\right)}=1-\frac{\left|\text{cor}(E_{I}^{\left(1\right)},E_{J}^{\left(1\right)})-\text{cor}(E_{I}^{\left(2\right)},E_{J}^{\left(2\right)})\right|}{2}.$$

Here $E_{I}^{\left(s\right)}$ denotes the eigengene of the I-th consensus module in data set *s*. High values of $Preserv_{IJ}^{\left(1,2\right)}$ indicate strong correlation preservation between eigengenes *I *and *J *across the two networks. The scaled connectivity *C*_*I*_(*Preserv*^(1,2)^) is given by

$$C_{I}(Preserv^{\left(1,2\right)})=1-\frac{\sum_{J\neq I}\left|\text{cor}(E_{I}^{\left(1\right)},E_{J}^{\left(1\right)})-\text{cor}(E_{I}^{\left(2\right)},E_{J}^{\left(2\right)})\right|}{2(N-1)},$$

and is close to 1 if the correlations between the *I*-th eigengene and the other eigengenes are preserved across the two networks. The density *D*(*Preserv*^(1,2)^) is given by

$$D(Preserv^{\left(1,2\right)})=1-\frac{\sum_{I}\sum_{J\neq I}\left|\text{cor}(E_{I}^{\left(1\right)},E_{J}^{\left(1\right)})-\text{cor}(E_{I}^{\left(2\right)},E_{J}^{\left(2\right)})\right|}{2N(N-1)}.$$

Larger values of *D*(*Preserv*^(1,2)^) indicate stronger correlation preservation between all pairs of eigengenes across the two networks. Measures (5, 6) are intuitive, descriptive measures for assessing the extent of preservation between networks. To arrive at a statistical significance level (*p*-value), one can use a permutation test (described in Methods). Many statistical tests have been proposed to test for differences between correlations, *e.g.*, [14-16].

### Application 1: Differential eigengene network analysis of human and chimpanzee brain expression data

Here we report results of our differential eigengene network analysis of human and chimpanzee microarray brain data. The microarray data were originally published in [17]. A gene co-expression analysis of these data is reported in [11]. To facilitate a comparison with the original marginal module analysis, we used the genes selected by that work. The data, R code, and more details of this analysis can be found in Additional File 1 and on our webpage.

To find consensus modules, we used the consensus dissimilarity measure (Eq. 22) and average linkage hierarchical clustering. Genes of a given consensus module were assigned the same color, while unassigned genes were labeled grey. We found 7 consensus modules, shown in Fig. 2A: black (41 genes), blue (40 genes), brown (294 genes), pink (41 genes), red (78 genes), turquoise (884 genes), and yellow (151 genes). The functional enrichment analysis of these consensus modules is described below. For each data set, we represented the consensus modules by their corresponding module eigengenes and constructed an eigengene network between them (Eq. 1).

The differential eigengene network analysis yields two main novel findings that could not have been obtained using a standard marginal method. First, we find that the *relationships *between the module eigengenes are highly preserved. Figs. 2E and 2H show the eigengene networks *A*_*Eigen*,*human *_and *A*_*Eigen*,*chimp*_, respectively. It is clear that the human and chimp eigengene networks of consensus modules are highly preserved. As described in Eq. (4), we defined a preservation network *Preserve*^*human*,*chimp *^= *Preserv*(*A*_*Eigen*,*human*_, *A*_*Eigen*,*chimp*_) between the 7 consensus eigengenes.

For each individual eigengene, we find that its relationships with the other eigengenes is highly preserved as reflected by a high connectivity in the preservation network (Eq. 5): *C*_*red*_(*Preserve*^*human*,*chimp*^) = 0.94, *C*_*black *_= 0.95, *C*_*yellow *_= 0.92, *C*_*turquoise *_= 0.95, *C*_*pink *_= 0.91, *C*_*blue *_= 0.91, *C*_*brown *_= 0.94. We find a high overall preservation (Eq. 6) between the two networks as reflected by a high density of the preservation network *D*(*Preserve*^*human*,*chimp*^) = 0.93. Figs. 2F,G summarize our findings about the relationships of the consensus modules.

The second novel finding is that the consensus eigengenes in the human data set fall into three branches (meta-modules), see Fig. 2C. The first meta-module consists of the red, black, and yellow eigengenes; the second meta-module contains the turquoise eigengene; and the third meta-module contains the pink, blue and brown eigengenes. Remarkably, these 3 meta-modules can also be detected in the chimp data, see Fig. 2D. While the definition of consensus modules trivially implies that they are preserved between the two data sets, it is a non-trivial result that in this application the meta-modules are preserved as well. 

To understand the biological meaning of the consensus modules, we studied differential expression of the consensus module eigengenes across the brain areas from which the microarray samples were taken. The results are summarized in Fig. 2 which shows the t-test *p*-values of differential expression of module eigengenes in the various brain regions from which samples were taken. Clearly, eigengenes can be characterized by their differential expression patterns in different brain regions. Furthermore, this analysis allows a biologically meaningful characterization of the meta-modules. The first meta-module (comprised of the black, yellow, and red module eigengenes) represents 270 genes that tend to be differentially expressed in the caudate nucleus. The second meta-module (comprised only of the turquoise eigengene) represents 884 genes that tend to be differentially expressed in cerebellum. The third meta-module (comprised of the pink, blue, and brown module eigengenes) represents 375 genes that are differentially expressed in the cortical samples. Thus, the meta-modules of this application correspond to biologically meaningful super-sets of modules and genes.

Given the strong relationships between modules in each meta-module, it is natural to ask whether the consensus modules are truly distinct. For example, the black and red modules show very similar levels of differential expression, see Fig. 2B. In this case, gene ontology information suggests that the two modules are indeed distinct. The black module is enriched with white matter related genes while no such enrichment can be found for the red module [11]. Likewise, gene ontology suggests that the yellow and black modules are distinct even though their module eigengenes are correlated.

In summary, the eigengene network analysis reveals a higher order organization of the consensus modules in the transcriptome.

#### Comparing our findings to a standard marginal module analysis

A standard approach for comparing the modules between several network is to identify modules in a 'reference' network and to study the preservation of the module assignment in the other networks [7]. In the original analysis, Oldham *et al *chose the human gene co-expression network as reference network since both preservation and non-preservation of human modules was of interest. This marginal module analysis is appropriate when the modules of one data set are the focus of the analysis but it is not designed to identify consensus modules that form the focus of our article. To compare differential eigengene network analysis analysis to the standard marginal module method, we compared our consensus modules to the 7 human modules found in [11]. We used a pairwise Fisher exact test to determine whether there is significant overlap between the consensus and the human modules. The results are summarized in Additional File 2. Overall, we find good agreement between consensus modules and human specific modules, which reflects the fact that most human modules are preserved in chimpanzees. Most of the human modules can be assigned to a consensus module and vice-versa, except for the human blue (360 genes) and green (126) modules which mostly disappeared from the consensus. Interestingly, small remnants (24 and 12 genes, respectively) of the two modules form the majority of the only consensus module (labeled pink, 41 genes) that does not have a clear human counterpart. Another small remnant (33 genes) of the human blue module forms most of the consensus blue module (40 genes).

The green and blue human modules were found to represent mostly cortical samples (and cerebellum for the green module) and were the least preserved in chimpanzees [11]. This is congruent with our finding of their lack of conservation using the consensus module method. One possible explanation for the absence of these modules in chimpanzees is that they largely reflect gene expression in the cerebral cortex, a brain region that has expanded dramatically in the human lineage. The standard marginal differential network analysis also identified several genes – LDOC1, EYA1, LECT1, PGAM2 – whose connectivities (Eq. 8) were significantly lower in the chimp network. None of these genes are present in our consensus modules, providing additional evidence of the method's agreement with the results of [11].

By definition, the consensus module detection is designed to find modules that are shared between data sets. Obviously, there will be many applications where data set specific modules are of interest. In such applications a standard marginal module detection analysis will be preferable.

### Application 2: Differential eigengene network analysis of four mouse tissues

**Figure 3:**
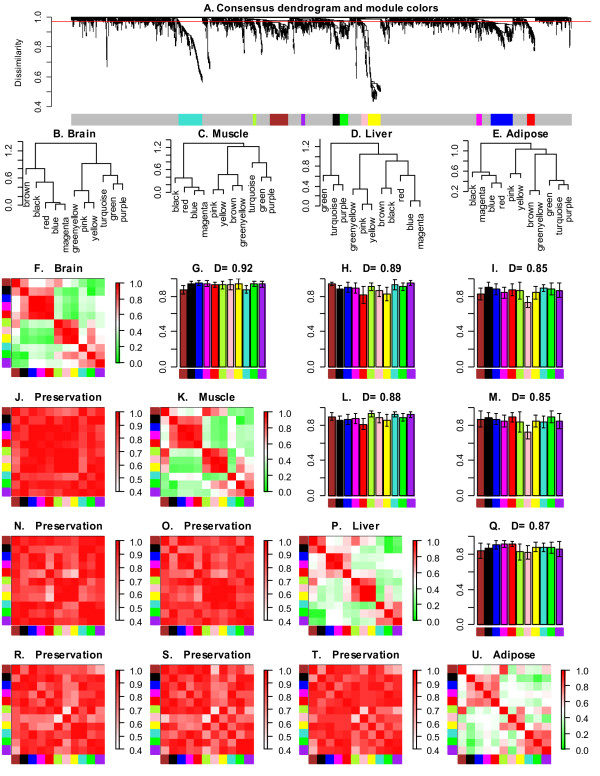
**Differential eigengene network analysis across four tissues in female mice**. A. Hierarchical clustering dendrogram of genes for identifying consensus modules (see text). Branches of the dendrogram, cut at the red line, correspond to consensus modules. Genes in each module are assigned the same color, shown in the color band below the dendrogram. Genes not assigned to any of the modules are colored grey. Biological significance of the found modules was assessed by functional enrichment analysis, presented in the main text and in Additional File 4. B.–E. Clustering dendrograms of consensus module eigengenes for identifying meta-modules. F.–U. Matrix of plots showing the consensus eigengene networks in the four tissues. Each row and column corresponds to one tissue as indicated on the diagonal plots. The diagonal plots F., K., P., U. show the heatmap plots of eigengene adjacencies in each eigengene network. Each row and column corresponds to one eigengene (labeled by consensus module color). Within each heatmap, red indicates hight adjacency (positive correlation) and green low adjacency (negative correlation) as shown by the color legend. Each of the upper triangle plots (G., H., I., L., M., Q.) shows a barplot of of preservation of relationships of consensus eigengenes, Eq. (16) between the two tissues (corresponding row and column) as well as the overall network preservation measure *D *for that pair of tissues, Eq. (17). The lower triangle plots (J., N., O., R., S., T.) show the adjacency heatmaps for the pairwise preservation networks of the tissues corresponding to the row and column, Eq. (15). In the heatmap, each row and column corresponds to a consensus module; saturation of the red color encodes adjacency according to the color legend.

We analyzed gene expression data obtained from female mice of an F2 mouse intercross [18]. The microarray data measured gene expression levels in four different mouse tissues: liver, brain, adipose and muscle. More details concerning the data are presented in Additional File 3 and on our webpage. The consensus dissimilarity (Methods, Eq. (22)) was used as input to average linkage hierarchical clustering. In the resulting dendrogram, consensus modules were identified by the Dynamic Tree Cut branch cutting method [19]. We found 11 consensus modules (Fig. 3A): black (50 genes), blue (149 genes), brown (125 genes), green (59 genes), green-yellow (25 genes), magenta (36 genes), pink (44 genes), purple (27 genes), red (55 genes), turquoise (162 genes) and yellow (87 genes). Functional enrichment analysis of these modules is presented below.

Figures 3F,K,P, and 3U show the eigengene networks *A*_*Eigen*,*brain*_, *A*_*Eigen*,*muscle*_, *A*_*Eigen*,liv*er*_, and *A*_*Eigen, adipose*_, respectively. To assess the preservation of consensus modules across pairs of tissues, we defined preservation networks (Eq. 15), *e.g.*, *Preserv*^*muscle*,*adipose *^= *Preserv*(*A*_*Eigen*,*muscle*_, *A*_*Eigen*,*adipose*_). We find the following overall preservation values between the eigengene networks: *D*(*Preserv*^*brain*,*muscle*^) = 0.93, *D*^*brain*,*liver *^= 0.88, *D*^*brain*,*adipose *^= 0.85, *D*^*muscle*,*liver *^= 0.88, *D*^*muscle*,*adipose *^= 0.85, *D*^*liver*,*adipose *^= 0.87. Hence, at the level of tissues, we observe good preservation between the consensus eigengene networks with highest preservation between the brain and muscle tissues. Interestingly, these two data sets also show the strongest relationships between the eigengenes in each data set (strongest red and green patterns in the heatmap plots). This can be measured by the density of the absolute values of ME correlations, *D*_cor _≡ *D*(|cor(*E*_*I*_, *E*_*J*_)|). For the muscle and brain network we find *D*_cor,*muscle *_= 0.45 and *D*_cor,*brain *_= 0.45. The eigengenes in liver show, as a data set, relationships somewhat similar to those of brain and muscle, though the patterns in the heatmap plot are not as strong, *D*_cor,*liver *_= 0.37. The adipose tissue shows the weakest relationships between the module eigengenes, *D*_cor,*adipose *_= 0.31. The eigengene preservations, *e.g.*, *C*_*red*_(*Preserve*^*muscle*,*adipose*^) can be found in Fig. 3, in the upper triangle of the matrix of plots F-U.

As an aside, we mention that pairwise network preservation measures are directly comparable only when the compared preservation networks involve the same set of consensus eigengenes, as is the case in this four-tissue application.

We find that the eigengene networks contain meta-modules, *i.e.*, groups of highly correlated eigengenes (Figs. 3B–E). As an example, we focus on the meta-modules in the brain eigengene network. As can be seen from Fig. 3, the consensus eigengenes in brain tissue form 3 meta-modules that are partially preserved in the other tissues. Specifically, the first brain meta-module consists of the black, blue, magenta, and red consensus eigengenes. It is highly preserved in muscle and adipose but less so in liver. The second brain meta-module consists of the green-yellow, pink and yellow consensus eigengenes. This meta-module is highly preserved in muscle and liver but less so in adipose. The third brain meta-module consists of the turquoise, green and purple eigengenes. It is highly preserved in liver and adipose but less so in muscle. These results show that meta-modules may or may not be preserved across the different eigengene networks.

To understand the biological meaning of the consensus modules, we used functional enrichment analysis using gene ontology information [20]. The detailed results including alternative methods for adjusting for multiple comparisons can be found in the functional enrichment table presented in Additional File 4. Overall, we find that most modules are significantly enriched with known gene ontologies. Specifically, the black module is highly enriched with ribosomal genes (Bonferroni-corrected Fisher's exact *p*-value *p *= 8 × 10^-10^); the blue module with immune/stimulus/defense response (*p *< 3 × 10^-17 ^for each of the three terms); brown with translation regulator activity (*p *= 4 × 10^-3^) and nucleotide binding (*p *= 5 × 10^-3^); magenta with stimulus/defense response (*p *< 2 × 10^-6^) and signal pathways (*p *< 2 × 10^-3^); red with cell cycle (*p *= 1.4 × 10^-19^) as well as nucleotide/ATP binding (*p *< 10^-8^); turquoise with protein binding (*p *= 6 × 10^-3^); yellow with carbohydrate metabolism (*p *= 3 × 10^-4^); pink and green-yellow with protein localization (*p *= 0.003 and *p *= 0.004), and green with alternative splicing/intracellular organelles (*p *= 4 × 10^-4^).

Our method detected two protein transport and localization modules (pink and green-yellow) and one may ask whether these modules are truly distinct. The two modules are closely related in 3 of the 4 data sets, but in the adipose tissue they have a weak (and negative) correlation of -0.24. Hence, from the consensus point of view, they are two distinct modules. Further, note that the green and black modules are very close on the consensus dendrogram, and their module eigengene (ME) correlation is high in absolute value but negative. The functional enrichment analysis suggests that the modules are different, although some terms are related (ribosomes for the black module and intracellular organelle for the green); this is an indication that the sign of the correlation of eigengenes is biologically meaningful.

While a standard marginal module analysis would succeed in studying preservation of individual data set modules, the consensus eigengene module analysis allows us to find shared modules and to study higher-order relationships between the consensus modules. Meta-modules in the brain tissues indicate the following relationships: the first (black, blue, magenta, red) suggests a relationship among ribosomal, immune/defense/stimulus response and cell cycle pathways; the second (green-yellow, pink, yellow) between protein localization and carbohydrate metabolism; the third (turquoise, green, purple) among protein binding and alternative splicing/intracellular organelle pathways.

The data also include clinical trait information on the mice (*e.g.*, cholesterol and insulin levels, body weight, etc.), and one can ask whether some of the consensus modules (or more precisely, their eigengenes) relate significantly to any of the traits. We find no significant correlation between consensus module eigengenes and the traits. In application 3, we report significant relationships between consensus modules and clinical traits.

#### Permutation test of consensus module membership

**Figure 4:**
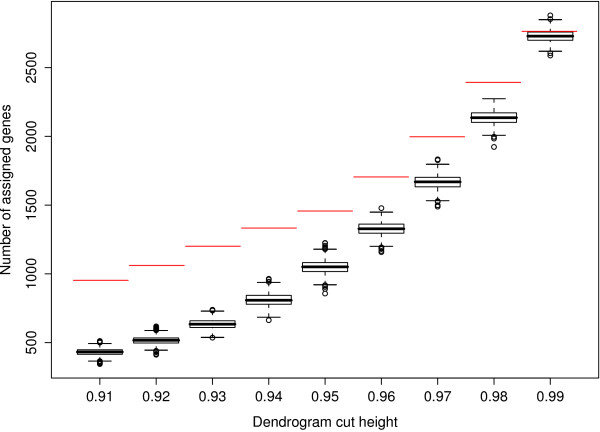
**Permutation test results for showing that the number of genes in consensus modules is highly significant**. Here we use the brain and muscle tissues of female mice. The size of a consensus module depends on the height cut-off used for cutting branches off the dendrogram. Thus, the number of genes in a consensus module (y-axis) depends on the height cut-off (x-axis). The red horizontal lines represent the observed number of genes in consensus modules for the original (unpermuted) data set. The boxplots (black) summarize the number of genes assigned to consensus modules after the gene list has been permuted between the two data set (1000 random permutations). For height cut-offs less than 0.99, the observed number of consensus genes is highly significant (*p *= 0.001).

We used the data from the brain and muscle tissues to perform a permutation test (described in Methods) of consensus module detection. We defined the combined number of genes assigned to consensus modules as test statistic. This test statistic was highly significant (*p *≤ 0.001), which shows that the number of genes in the consensus modules was highly significant. However, this results depends on the level of stringency for defining consensus modules. Fig. 4 shows that as the height cutoff for the detection of branches in the consensus dendrogram increases, the probability of finding spurious consensus modules (and genes therein) increases; for excessively high branch cutoffs levels, the probability of finding as many genes in permuted data sets as in the unpermuted becomes unacceptably high.

### Application 3: Consensus modules across female and male mouse liver tissues

**Figure 5:**
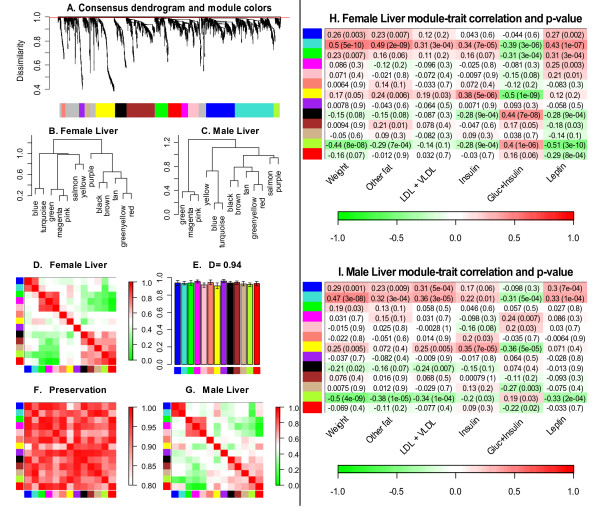
**Differential eigengene network analysis across female and male mouse liver tissues**. A. Hierarchical clustering dendrogram of genes for identifying consensus modules (see text). Branches of the dendrogram, cut at the red line, correspond to consensus modules. Genes in each module are assigned the same color, shown in the color band below the dendrogram. Genes not assigned to any of the modules are colored grey. B.–C. Clustering dendrograms of consensus module eigengenes for identifying meta-modules. D.–G. Matrix of plots showing the consensus eigengene networks. The diagonal plots D., G. show heatmap plots of eigengene adjacencies in each eigengene network. Each row and column corresponds to one eigengene (labeled by consensus module color). Within each heatmap, red indicates high adjacency (positive correlation) and green low adjacency (negative correlation) as shown by the color legend. E. Barplot of preservation of relationships of consensus eigengenes between the two data sets, Eq. (16), as well as the overall network preservation measure *D*, Eq. (17). Each colored bar corresponds to the eigengene of the corresponding color. The height of the bar (*y*-axis) gives the eigengene preservation measure (16). F. Adjacency heatmap for the preservation network between female and male consensus eigengene networks, Eq. (15). Each row and column corresponds to a consensus module; saturation of the red color encodes adjacency according to the color legend. H., I. Consensus module significance for clinical traits, given by the correlation of the corresponding module eigengene (row) with the clinical trait (column). Shown are correlations and *p*-values; cell color encodes correlation (red, positive correlation, green, negative correlation according to the color legend).

Here we apply the differential eigengene network analysis to liver expression data from female and male mice of the above-mentioned F2 mouse intercross. The consensus module detection method identified 11 consensus modules, shown Fig. 5A: black (182 genes), blue (444 genes), brown (439 genes), green (207 genes), green-yellow (82 genes), magenta (105 genes), pink (168 genes), purple (83 genes), red (203 genes), salmon (58 genes), tan (67 genes), turquoise (605 genes), and yellow (302 genes). Overall, there is excellent preservation between the female and male eigengene networks, *D*(*Preserv*^*female*,*male*^) = 0.94 (Figs. 5E,F). The module eigengene dendrograms in Figs. 5B,C as well as at the eigengene network heatmaps in Figs. 5D,G indicate that the two data sets share three meta-modules. The first one contains the blue and turquoise modules (1049 genes), the second one contains the green, magenta and pink modules (480 genes), and the third one contains the black, brown, tan, green-yellow and red modules (466 genes).

The experimental data include clinical traits such as mouse body weight, cholesterol levels, etc. As detailed in Additional File 5, we selected 7 potentially interesting traits. Figs. 5H,I present the correlations and corresponding *p*-values for relating the clinical traits to the module eigengenes. We find that the turquoise module (605 genes) is highly significantly correlated with weight in both the female (*r *= 0.5, *p *= 5 × 10^-8^) and male samples (*r *= 0.47, *p *= 3.1 × 10^-8^). The greenyellow module (82 genes) relates to weight with comparable correlations, *r *= -0.44 (*p *= 8 × 10^-8^) and *r *= -0.50 (*p *= 4 × 10^-9^) in females and males, respectively. The yellow module is significantly related to insulin levels in both the female and male data sets, *r *= 0.38 (*p *= 5 × 10^-6^) and *r *= 0.35 (*p *= 7 × 10^-5^), respectively. The correlation between the eigengenes of the consensus turquoise and greenyellow modules are -0.68 and -0.74 in the female and male samples, respectively; the module eigengenes are relatively close by absolute value of the correlation, but the sign difference suggests that they distinct. This result is another motivation to use signed networks (Eq. 1) to describe the relationships between eigengenes.

Given that the female and male networks appear similar but not the same, one may ask whether the consensus module analysis provides an indication of how they differ. For this purpose we compared the female liver module assignment as reported in [18] to our consensus module assignment, see Additional File 6. Using the same parameters for the clustering and branch detection, we found that two of the 12 modules (labeled by salmon and light-yellow color) in that work are not represented in the consensus modules. Investigating the function of these two modules is beyond the scope of this work.

### Simulation studies of consensus modules

To assess the performance of the consensus module detection method, we performed a simulation study involving two simulated gene expression data sets. The two data sets contained both shared and non-shared modules. The actual simulation procedure is described in more detail in Additional File 7 and the R code can be found on our webpage.

Briefly, each simulated module is built around a chosen seed profile (referred to as the true module eigengene) by adding gene expression profiles with increasing amount of noise. We studied the performance of consensus module detection under varying levels of added noise. The sensitivity and specificity are determined from the numbers of true and false positives (*n*_*TP *_and *n*_*FP*_) and true and false negatives (*n*_*TN *_and *n*_*FN*_) as Sensitivity = *n*_*TP*_*/*(*n*_*TP *_+ *n*_*FN*_), Specificity = *n*_*TN*_*/*(*n*_*TN *_+ *n*_*FP*_). To measure the fidelity of the calculated module eigengenes to the true module eigengenes, we report the proportion *P*_0.95 _of the detected modules whose eigengene has a correlation greater than 0.95 with the true module eigengene, *i.e.*, Fidelity = *P*_0.95_. Results of the simulation are  summarized in Table 1. We found that when noise is low and modules are very clearly defined, the sensitivity, specificity, and fidelity are 100%. It is worth noting that for low and moderate noise levels, the fidelity does not vary substantially with changes in the branch cut height, indicating that module eigengenes are robust to inclusion/exclusion of moderate numbers of genes in the module. As the noise increases, sensitivity, specificity, and fidelity decrease. We note that the specificity and sensitivity depend on the choice of cutting parameters for the cluster trees. We have not performed an exhaustive search to identify parameter values that would give optimal performance. Our default settings perform well across a range of different simulation models.

## Discussion

We propose the use of eigengene networks to study the relationships between co-expression modules. Eigengene networks will be useful for any module detection method that leads to modules of highly correlated genes. While eigengene networks can easily be adapted to other co-expression module detection methods, we define them within the framework of weighted gene co-expression network analysis since this framework preserves the continuous nature of the co-expression information and leads to robust results [4,7]. Our empirical applications illustrate the kind of novel questions that can be addressed with eigengene networks. We find that modules can be organized into meta-modules that can be biologically meaningful and interesting.

Eigengene networks can naturally be integrated with other types of quantitative data. For example a microarray sample trait *T *(such as body weight or survival time) can be included as an additional node of the eigengene network. The adjacency between an eigengene *E*_*I *_and the sample trait *T *can be defined as *a*_*Eigen*,*I*,*T *_= (1 + cor(*E*_*I*_, *T*))/2, generalizing Eq. (24) (Methods). Eigengenes that are adjacent to a clinical trait may correspond to pathways (modules) that are associated with the clinical trait. We illustrate this point in our third application involving female and male mouse liver data, in which we analyze the relationship between consensus modules and clinical traits.

Eigengene networks can be used for describing module relationships in a single data set (single eigengene network analysis) or they can be used to compare module relationships across different data sets (differential eigengene network analysis). To facilitate differential eigengene network analysis, we propose methods for finding consensus modules. Our approach for detecting consensus modules relies on a consensus dissimilarity measure (Methods, Eq. 22) that compares topological overlap matrices of different data sets. In our applications, consensus implies that the modules are present in all data sets (networks). In other applications, it may be preferable to relax this stringent requirement and look for 'common' modules instead. For example, if the number of studied data sets is large (say more than 5), the robustness can be increased by replacing the minimum by a suitably chosen quantile (*e.g.*, the median). Details of such a generalization are presented in Methods.

Since we define the consensus topological overlap as a minimum (Methods, Eq. 18), a bias will result if the topological overlap of one network tends to be higher (or lower) than that of the other data sets because of non-biologic reasons including different microarray platforms, gene expression normalization methods, or different sample sizes. To address this potential bias, one can scale the individual topological overlap matrices or adjacency matrices. Alternatively, we describe a highly robust but less sensitive method for defining consensus modules in the Methods (Eq. 37). In brief, this robust method defines modules in each of the individual data sets and defines consensus modules by keeping track of shared module membership. Module detection depends on several parameters choices, *e.g.*, how to cut off branches of a hierarchical cluster tree. In practice, it is advisable to carry out a robustness analysis with regard to the module definition. For example, the reader can use the R code published on our web page to verify that our findings are relatively robust. Since the module eigengene (first principal component) represents a suitably defined average gene expression profile, it is highly robust with regard to moderate changes in module membership. We find that the consensus eigengene network construction is highly robust and it has high sensitivity and specificity in our simulation studies.

## Conclusion

We find that eigengene network methods lead to mathematically robust and biologically meaningful results. We provide three microarray data applications illustrating that eigengene networks effectively represent module relationships. Studies of inter-modular relationships may reveal changes in pathway dependencies due biological perturbations.

## Methods

### Network adjacency matrices and connectivity

We consider networks that are fully specified by an *n *× *n *symmetric adjacency matrix *A *= (*a*_*ij*_). For an *unweighted *network, the adjacency *a*_*ij *_equals 0 if nodes *i *and *j *are not connected and 1 if the nodes are connected. For a *weighted *network 0 ≤ *a*_*ij *_≤ 1 reports the connection strength between nodes *i *and *j*. As a convention, we set the diagonal elements to 1, *i.e.*, *a*_*ii *_= 1. In summary, we study networks whose adjacencies satisfy the following conditions

$$\begin{array}{c} 0\leq a_{ij}\leq 1,\\ a_{ij}=a_{ji},\\ a_{ii}=1. \end{array}$$

Since we study gene co-expression networks, we usually refer to the network nodes as 'genes'. For the *i*-th gene, the *scaled connectivity *(also referred to as scaled degree) is defined as

$$C_{i}(A)\equiv \frac{\sum_{j\neq i}a_{ij}}{n-1}.$$

Note that 0 ≤ *C*_*i*_(*A*) ≤ 1. Genes with high connectivity are sometimes referred to as 'hub' genes. The *network density D*(*A*) is defined as the average scaled connectivity [21],

$$D(A)\equiv \frac{1}{n}\sum_{i}C_{i}=\frac{\sum_{i}\sum_{j\neq i}a_{ij}}{n(n-1)}.$$

Note that 0 ≤ *D*(*A*) ≤ 1. The density equals the average adjacency (connection strength) between the genes.

### Transformations of the adjacency matrix

We find it useful to introduce the following transformations that map an *n *× *n *adjacency matrix to another *n *× *n *matrix. The power transformation *Power*(*A*, *β*) raises each adjacency to a fixed power *β*, *i.e.*,

$$Power_{ij}(A,\beta )\equiv a_{ij}^{\beta }.$$

Note that *Power*(*A*, *β*) also satisfies the conditions of an adjacency matrix (Eq. 7). By choosing a power *β *> 1 the power transformation can be used to emphasize large adjacencies at the expense of low ones, *i.e.*, the power transformation can be used for 'soft-thresholding' [4]. We use this approach for defining weighted gene co-expression networks.

The topological overlap transformation *TOM*(*A*) replaces each adjacency *a*_*ij *_by a normalized count neighbors that are shared by the nodes *i*, *j*. In an unweighted network, the number of shared neighbors of genes *i *and *j *is given by ∑_*u*≠*i*,*j*_*a*_*iu*_*a*_*ju*_. For a weighted network *A*, the topological overlap measure (TOM) is defined as

$$TOM_{ij}(A)\equiv \frac{\sum_{u\neq i,j}a_{iu}a_{uj}+a_{ij}}{\min(\sum_{u\neq i}a_{iu},\sum_{u\neq j}a_{ju})+1-a_{ij}}.$$

One can show that *TOM*_*ij*_(*A*) also satisfied the conditions (Eq. 7) of an adjacency matrix [4,21]. The topological overlap of two genes reflects their similarity in terms of the commonality of the genes they connect to. The TOM transformation can lead to a more robust network and larger modules [22-24]. The quantile transformation

$$Quant_{q,ij}\left(A^{\left(1\right)},A^{\left(2\right)},...\right)=quantile_{q}(a_{ij}^{\left(1\right)},a_{ij}^{\left(2\right)},...)$$

takes multiple adjacency matrices of the same dimension as input and yields a single adjacency matrix whose component *Quant*_*q*,*ij *_is the *q*-th quantile of the corresponding components $a_{ij}^{\left(1\right)},a_{ij}^{\left(2\right)},...$ of the input matrices. Two special cases are of particular interest, the *Min *= *Quant*_*q*=0 _and *Max *= *Quant*_*q*=1 _transformations,

$$\begin{array}{lll} Min_{ij}\left(A^{\left(1\right)},A^{\left(2\right)},...\right) & = & \min\left(a_{ij}^{\left(1\right)},a_{ij}^{\left(2\right)},...\right), \end{array}$$

$$\begin{array}{ccc} Max_{ij}\left(A^{\left(1\right)},A^{\left(2\right)},...\right) & = & \max\left(a_{ij}^{\left(1\right)},a_{ij}^{\left(2\right)},...\right). \end{array}$$

#### Preservation Network

The preservation transformation *Preserv*(*A*^(1)^, *A*^(2)^,...) can be used to determine whether adjacencies are preserved between given networks *A*^(1)^, *A*^(2)^,.... Specifically,

*Preserv*_*ij*_(*A*^(1)^, *A*^(2)^,...) = 1 - [*Max*_*ij*_(*A*^(1)^, *A*^(2)^,...) - *Min*_*ij*_(*A*^(1)^, *A*^(2)^,...)].

We use this transformation in our differential network analysis. Often we use the abbreviation $Preserv_{ij}^{\left(1,2,...\right)}$ = *Preserv*_*ij*_(*A*^(1)^, *A*^(2)^,...). The closer $Preserv_{ij}^{\left(1,2,...\right)}$ is to 1, the better preserved is the adjacency between genes *i *and *j *across all of the compared networks. Note that *Preserv*^(1,2,...) ^satisfies our conditions of an adjacency matrix (Eq. 7) and we refer to it as the *preservation network*. The scaled connectivity of the preservation network,

$$C_{i}(Preserv^{\left(1,2\right)})=1-\frac{\sum_{j\neq i}\left|a_{ij}^{\left(1\right)}-a_{ij}^{\left(2\right)}\right|}{n-1},$$

is an aggregate measure of adjacency preservation for the *i*-th gene. The density of the preservation network,

$$D(Preserv^{\left(1,2\right)})=1-\frac{\sum_{i}\sum_{j\neq i}\left|a_{ij}^{\left(1\right)}-a_{ij}^{\left(2\right)}\right|}{n(n-1)},$$

is an aggregate measure of adjacency preservation between networks *A*^(1) ^and *A*^(2)^.

#### Consensus networks

We now introduce the notion of a consensus network for given input adjacency matrices *A*^(1)^, *A*^(2)^,.... Intuitively, two nodes should be connected in a consensus network only if all of the input networks 'agree' on that connection. This naturally suggest to define

*Consensus*_*ij*_(*A*^(1)^, *A*^(2)^,...) = *Min*_*ij*_(*A*^(1)^, *A*^(2)^,...).

The *Consensus *transformation is related to *Preserv *(Eq. 15): if the *Max*(*A*^(1)^, *A*^(2)^,...) network is dense, that is if *Max*_*ij*_(*A*^(1)^, *A*^(2)^,...) ≈ 1 for all pairs of nodes *i*, *j*, we find *Preserv*(*A*^(1)^, *A*^(2)^,...) ≈ *Consensus*(*A*^(1)^, *A*^(2)^,...). On the other hand, if the *Max *network is sparse with most adjacencies close to zero, *Preserv *and *Consensus *differ.

We use the definition (18) in all our applications, but generalizations are of interest as well. Our definition of the *Consensus *adjacency may be too stringent when dealing with more than a handful of networks. To address this, we use the quantile transformation (Eq. 12) to define a more robust consensus network as follows

*Consensus*_*q*,*ij*_(*A*^(1)^, *A*^(2)^,...) = *Quant*_*q*,*ij*_(*A*^(1)^, *A*^(2)^,...).

Note that our *Consensus *network (Eq. 18) is a special case of Eq. (19) with *q *= 0. For *q *= 0.25 and *q *= 0.5, the resulting consensus network is defined as the first quartile and the median, respectively, of the input adjacencies.

#### Dissimilarity transformation for module detection

The dissimilarity transformation *Dissim*(*A*) turns an adjacency matrix (which is a measure of similarity) into a measure of *dissimilarity *by subtracting it from 1, *i.e.*,

*Dissim*_*ij*_(*A*) ≡ 1 - *a*_*ij*_.

This transformation is useful for defining module detection procedures. As an aside, we mention that *Dissim*(*A*) does not satisfy our definition of an adjacency matrix since its diagonal elements equal 0.

### Module detection using hierarchical clustering

Many possible approaches for defining network modules have been proposed in the literature [25-29]. We define modules as clusters that result from using a pairwise node dissimilarity *d*_*ij *_as input of average linkage hierarchical clustering. For a gene network with adjacency matrix *A*, we use the topological overlap based dissimilarity

$$\begin{array}{ccc} d_{ij} & = & Dissim_{ij}(TOM(A))\\ & = & 1-\frac{\sum_{u\neq i,j}a_{iu}a_{uj}+a_{ij}}{\min(\sum_{u\neq i}a_{iu},\sum_{u\neq j}a_{ju})+1-a_{ij}}. \end{array}$$

This dissimilarity is used as input to average linkage hierarchical clustering. Branches in the resulting cluster tree (dendrogram) are referred to as modules. As detailed in our R tutorials, we use two different branch cutting techniques: the constant-height cut method and the dynamic tree cut method [19]. This module detection approach has been successfully used in several studies [6,7,11,18,22,30].

### Consensus modules

Consensus modules are defined as modules in the consensus network (Eq. 18). Analogously to the single network case (Eq. 21), we define a *consensus gene dissimilarity*

*Dissim*(*Consensus*(*TOM*(*A*^(1)^), *TOM*(*A*^(2)^),...)),

and use it as input to average linkage hierarchical clustering. Consensus modules are defined as branches of the resulting clustering tree. By definition, consensus modules consist of genes that are closely related in all networks *A*^(1)^, *A*^(2)^,...; in other words, the modules are present in all networks.

### Weighted gene co-expression network construction and module detection

Denote the *i*-th gene expression profile (where *i *= 1...*n*) across *m *microarrays as *x*_*i*_. Thus, *x*_*i *_is a vector with *m *components. We use two different measures of co-expression similarity to compare a pair of gene expression profiles *x*_*i *_and *x*_*j*_. The first measure *S *= (*s*_*ij*_) is the absolute value of the Pearson correlation coefficient, *i.e.*,

*s*_*ij *_= |cor(*x*_*i*_, *x*_*j*_)|

The second measure *S*_*signed *_is a linear transformation of the correlation that retains its sign:

$$s_{signed,ij}=\frac{1+\text{cor}(x_{i},x_{j})}{2}.$$

Note that *s*_*signed*,*ij *_equals 1, 1/2, and 0 if the correlation equals 1, 0, and -1, respectively.

The co-expression similarities are transformed into a weighted gene co-expression network [4,7] using the power transformation (Eq. 10):

$$\begin{array}{ccc} a_{weighted,ij} & = & Power_{ij}(S,\beta )=s_{ij}^{\beta }, \end{array}$$

$$\begin{array}{ccc} a_{signed,ij} & = & Power_{ij}(S_{signed},\beta )=s_{signed,ij}^{\beta }. \end{array}$$

The power transformation with *β *> 1 allows one to suppress low co-expression similarities that may be spurious while at the same time preserving the continuous nature of co-expression information.

In our applications, we use the unsigned adjacency (Eq. 25) to define gene co-expression networks. To choose the power *β*, we use the scale free topology criterion [4]. We define eigengene networks using the signed adjacency (Eq. 26) because we find it useful to preserve the sign of the co-expression information between module eigengenes. The scaled connectivity *C*_*i*_(*A*_*signed*_) is close to 1 and 0 if the correlations between *x*_*i *_and other network genes tend to be positive and negative, respectively.

An important step in network analysis is to identify modules of co-expressed genes. As detailed above, we define modules as clusters of genes with high topological overlap (Eq. 11) since this yields relatively large and robust modules [4,6,7,22,24].

### Module eigengenes

To define the module eigengene of a module, we use the Singular Value Decomposition (SVD) of the module expression matrix [31]. The gene expression matrix of the *I*-th module is denoted by *X*^(*I*) ^= ($x_{il}^{\left(I\right)}$), where the index *i *= 1, 2,...,*n*_*I *_corresponds to the module genes and the index *l *= 1, 2,...,*m *corresponds to the microarray samples. We assume that each gene expression profiles $x_{i}^{\left(I\right)}$, i.e. each row of *X*^(*I*)^, has been standardized to mean 0 and variance 1. The singular value decomposition of *X*^(*I*) ^is denoted by

*X*^(*I*) ^= *UDV*^*T*^,

where the columns of the orthogonal matrices *U *and *V *are the left- and right-singular vectors, respectively. Specifically, *U*^(*I*) ^is an *n*^(*I*) ^× *m *matrix with orthonormal columns, *V*^(*I*) ^is an *m *× *m *orthogonal matrix, and *D*^(*I*) ^is an *m *× *m *diagonal matrix of the singular values {|$d_{l}^{\left(I\right)}$|}. The matrices *V*^(*I*) ^and *D*^(*I*) ^are given by

$$\begin{array}{lll} V^{\left(I\right)} & = & (\begin{array}{cccc} v_{1}^{\left(I\right)} & v_{2}^{\left(I\right)} & \cdots & v_{m}^{\left(I\right)}), \end{array}\\ D^{\left(I\right)} & = & diag\{|d_{1}^{\left(I\right)}|,|d_{2}^{\left(I\right)}|,...,|d_{m}^{\left(I\right)}|\}. \end{array}$$

We assume that the singular values |$d_{l}^{\left(I\right)}$| are arranged in non-increasing order. Adapting terminology from [11,12,18,31], we refer to the first column of *V*^(*I*) ^as the *Module Eigengene*:

$$E^{\left(I\right)}=v_{1}^{\left(I\right)}.$$

An equivalent definition can be given in terms of principal component analysis where the module eigengene is defined as the first principal component. Since the orientation (*i.e.*, sign) of each singular vector is undefined, we fix the orientation of each eigengene by constraining it to have a positive correlation with the average gene expression across module genes. In practice, we find that the module eigengene explains typically more than 50 percent of the variance of the module expressions.

#### Relating genes within a module to the module eigengene

Although our approach emphasizes modules (represented by eigengenes) as the basic building blocks of eigengene networks, it is often important to have a measure of how closely related a particular *actual gene *is to the eigengenes Within the co-expression networks, a natural measure is the *eigengene-based connectivity k*_*E*_(*i*), defined as the correlation between the expression profile of the studied gene (denoted *x*_*j*_) and the eigengene *E*_*I*_,

$$k_{E_{I}}(j)=\text{cor}(x_{j},E_{I}).$$

The closer $k_{E_{I}}(j)$ is to 1 or -1, the stronger the evidence that the j-th gene is part of the *I*-th module.

### Definition of meta-modules in eigengene networks

Once an eigengene network is defined (Eq. 1), a module detection procedure can be applied to the eigengene networks. We refer to modules in eigengene networks as meta-modules. Because eigengene networks are orders of magnitude smaller than the original gene co-expression networks, we do not use use topological overlap based dissimilarity for finding meta-modules. Instead, we use the following dissimilarity

$$Dissim_{IJ}(A_{Eigen})=\frac{1-\text{cor}(E_{I},E_{J})}{2}.$$

Using this dissimilarity as input to average linkage hierarchical clustering leads to a cluster tree of modules (represented by eigengenes). The branches of this tree correspond to meta-modules in our applications.

#### Consensus meta-modules

Applying the concept of consensus to eigengene networks leads to the definition of consensus dissimilarity for eigengene networks,

$$Dissim(Consensus(A_{Eigen}^{\left(1\right)},A_{Eigen}^{\left(2\right)},...)).$$

This dissimilarity is used to define and detect consensus meta-modules, that is meta-modules present in all input eigengene networks. A small consensus dissimilarity between two eigengenes is an indication that the modules are closely related in all studied data sets. This may be due to biological reasons, namely when corresponding modules represent distinct but interacting pathways. On the other hand, the corresponding modules could also be non-distinct and should be merged. For example, the module detection method may have been too sensitive, which results in many but related modules. To decide whether close modules should be merged, we suggest to use external information, *e.g.*, gene ontology information, or to study module preservation in an independent data set.

#### Generalized consensus networks

A limitation of consensus networks defined by Eq. (18) (as well as by Eq. 19) and the consensus dissimilarity (22) is that the direct comparison of networks is only meaningful if the corresponding adjacencies have similar distributions. This need not be the case: differences in sample sizes, array platforms, or gene expression normalization methods may seriously bias the results of the Quantile transformation (Eq. 12). To address this, we propose a robust approach to defining consensus modules. The idea is to replace the *TOM*^(1)^, *TOM*^(2)^,... matrices in (22) by 'compressed' adjacency matrices $A_{compressed}^{\left(1\right)},A_{compressed}^{\left(2\right)},...$, defined using the following steps. First, module detection is performed in each dataset separately, and corresponding module eigengenes are calculated. In data set *s*, denote by *Module*^(*s*)^(*i*) the index of the module that gene *i *belongs to. *Module*^(*s*)^(*i*) may encode the original module membership or it can be defined using the module eigengene based connectivity measure (Eq. 30): each gene is assigned to the module for which it has the maximum module eigengene based connectivity: 

*             Module*^(*s*)^(*i*) = *argmax*_*J*_(|$k_{E_{J}}(i)$|). 

If gene *i *has not been assigned to any of the modules, define *Module*^(*s*)^(*i*) = 0. The "compressed" gene adjacency $a_{compressed,ij}^{\left(s\right)}$ for genes *i *and *j *in data set *s *is defined as the eigengene network adjacency of the corresponding eigengenes:

$$a_{compressed,ij}^{\left(s\right)}\equiv \{\begin{array}{ll} a_{Eigen,Module^{\left(s\right)}(i),Module^{\left(s\right)}(j)}^{\left(s\right)} & \text{for }Module^{\left(s\right)}(i)\neq 0\text{ and }Module^{\left(s\right)}(j)\neq 0\\ 0 & \text{for }Module^{\left(s\right)}(i)=0\text{ or }Module^{\left(s\right)}(j)=0 \end{array}$$

Recall that we define the eigengene adjacency as

$$a_{Eigen,IJ}^{\left(s\right)}=\frac{1}{2}\left[1+\text{cor}\left(E_{Module^{s}\left(i\right)}^{\left(s\right)},E_{Module^{s}\left(j\right)}^{\left(s\right)}\right)\right].$$

Thus, for *Module*^(*s*)^(*i*) ≠ 0, *Module*^(*s*)^(*j*) ≠ 0, the compressed adjacency is

$$a_{compressed,ij}^{\left(s\right)}=\frac{1}{2}\left[1+\text{cor}\left(E_{Module^{\left(s\right)}(i)}^{\left(s\right)},E_{Module^{\left(s\right)}(j)}^{\left(s\right)}\right)\right].$$

The generalized consensus network is defined as the consensus of the compressed similarities,

$$A_{generalized\;consensus}=Consensus(a_{compressed}^{\left(1\right)},a_{compressed}^{\left(2\right)},...).$$

For module detection, one could use a corresponding consensus gene dissimilarity

$$\begin{array}{lll} d_{ij} & = & Dissim_{ij}(Consensus(a_{compressed}^{\left(1\right)},a_{compressed}^{\left(2\right)},...))\\ & = & 1-min(a_{compressed,ij}^{\left(1\right)},a_{compressed,ij}^{\left(2\right)},...). \end{array}$$

As an aside, we mention that one could also use the quantile consensus *Consensus*_*q *_method (Eqs. 36, 37) instead of our minimum based consensus transformation. 

Our definition (Eq. 37) is quite intuitive: the consensus dissimilarity is zero for two genes that belong to the same module in every individual set; the consensus dissimilarity will be small if the two genes belong to closely related modules in each data set; and for a gene outside any properly defined module (colored in grey in our applications), the dissimilarity with any other gene will attain its maximum value of 1. A potential major advantage of definition (Eq. 37) is that the individual dataset modules need not be obtained using the same module detection procedure. This allows finding consensus modules in datasets whose properties differ substantially. The differences can be countered by using appropriate data set specific module detection methods. However, this freedom of choice greatly increases the parameters used in the consensus module detection, and as a result, increases the danger of over-fitting. In contrast, our minimum-based consensus TOM method, Eq (22) involves only one clustering tree and hence involves far fewer parameters.

### Permutation tests

To assess whether the number of genes in consensus modules is significant, we perform a permutation test. The test statistic can be chosen as the total number of genes assigned to consensus modules or it can be based on module sizes (*e.g.*, the minimum consensus module size). First, the test statistic is evaluated on the original, unpermuted data. This results in the 'observed' test statistic. Next, the statistic is evaluated on permuted versions of the data. To noise up any relationship between the modules of different data sets, the gene labels of at least one data set are randomly permuted. Next the consensus module detection is applied to the permuted data sets and the test statistic is evaluated. This procedure is repeated multiple (*e.g.*, 1000) times. By counting how often the observed test statistic exceeds the permuted test statistic, one can estimate a permutation test *p*-value. In all of our applications, the number of genes found in consensus modules is highly significant (*p *≤ 0.001).

## Availability and requirements

Project name: Consensus Eigengene Networks

Project home page: 

Operating system(s): Platform independent

Programming language: R

Licence: GNU GPL

## Authors' contributions

Both authors jointly developed the methods and wrote the article. PL implemented the methods and applied them to the data. Both authors read and approved the final manuscript.

**Table 1:** Simulation studies of consensus module detection.

Noise level	Branch cut	Consensus module detection		
		Sensitivity	Specificity	Fidelity
1	0.965	1	1	0.989
1	0.975	1	1	0.988
1	0.985	1	1	0.985
1	0.995	1	1	0.965

2	0.965	0.966	1	0.964
2	0.975	0.984	1	0.958
2	0.985	0.998	1	0.949
2	0.995	1	1	0.935

3	0.965	0.717	1	0.871
3	0.975	0.823	1	0.838
3	0.985	0.929	1	0.824
3	0.995	0.997	0.999	0.822

4	0.965	0.457	1	0.823
4	0.975	0.589	1	0.744
4	0.985	0.739	0.997	0.713
4	0.995	0.928	0.995	0.675

5	0.965	0.0753	1	0.636
5	0.975	0.16	1	0.421
5	0.985	0.296	0.992	0.415
5	0.995	0.643	0.966	0.363

6	0.965	0.00345	1	0.667
6	0.975	0.0138	1	0.333
6	0.985	0.077	0.971	0.209
6	0.995	0.355	0.954	0.168

Using simulated data to assess the performance of the consensus module detection method. The column 'noise level' reflects the amount of noise added to the simulated data (details can be found in Additional File 7). The modules were defined as branches of an average linkage hierarchical cluster tree. The column 'branch cut' reports the heights used for cutting branches of the cluster tree. Sensitivity, specificity and fidelity are defined in the text.

## Supplementary Material

Additional file 1 **Detailed description of data for Application 1, differential eigengene analysis of human and chimpanzee brain expression data**. This document describes the human and chimp microarray data sets and the module detection method. The R code posted on our web page allows one to reproduce the Figures and tables reported in the main text. Click here for file

Additional file 2 **Comparing human-chimp consensus modules to their human data set specific counterparts**. This document describes a comparison between our human-chimp consensus modules and the human-specific modules detected by Oldham *et al *[11]. Click here for file

Additional file 3 **Detailed description of data for Application 2, differential eigengene network analysis of expression data from four tissues in female mice**. This document describes the mouse tissue microarray data sets and the module detection method. The R code posted on our web page allows one to reproduce the Figures and tables reported in the main text. Click here for file

Additional file 4 **Functional enrichment analysis of consensus modules across four tissues of female mice**. In this document we report functional enrichment *p*-values provided by the online tool DAVID [20]. The results show that our consensus modules are highly significantly enriched with known gene ontologies. Click here for file

Additional file 5 **Detailed description of data for Application 3, differential eigengene network analysis between female and male mice**. In this document we describe the details of female and male mouse liver data and the differential eigengene network analysis. We analyze the relationship between consensus modules and clinical traits. The R code posted on our web page allows one to reproduce the Figures and tables reported in the main text. Click here for file

Additional file 6 **Comparing female-male mouse liver consensus modules to their female data set specific counterparts**. This document describes a comparison between our male-female mouse consensus modules and the female liver-specific modules detected by Ghazalpour *et al *[18]. Click here for file

Additional file 7 **Simulation of gene expression data sets**. This document describes the simulation of gene expression data sets with a modular structure. The simulation R code can be found on our web page. Click here for file
