# Supplementary material for: Eigengene networks for studying the relationships between co-expression modules
Source: BMC Syst Biol. 2007 Nov 21;1:54. doi: 10.1186/1752-0509-1-54 (PMC2267703; doi:10.1186/1752-0509-1-54)
Supplement: Additional file 1 — Detailed description of data for Application 1, differential eigengene analysis of human and chimpanzee brain expression data. This document describes the human and chimp microarray data sets and the module detection method. The R code posted on our web page allows one to reproduce the Figures and tables reported in the main text. [file 1752-0509-1-54-S1.DOC]

**Eigengene Network Analysis of**

**Human and Chimpanzee Microarray Data**

**R Tutorial**

Peter Langfelder and Steve Horvath

Correspondence: [shorvath@mednet.ucla.edu](mailto:shorvath@mednet.ucla.edu), [Peter.Langfelder@gmail.com](mailto:Peter.Langfelder@gmail.com)

The methods and biological implications are described in the following reference

- *Langfelder P, Horvath S (2007) Eigengene networks for studying the relationships between co-expression modules. BMC Bioinformatics*

The microarray data and processing steps are described in

- *Oldham M, Horvath S, Geschwind D (2006) Conservation and Evolution of Gene Co-expression Networks in Human and Chimpanzee Brains. PNAS. Nov 21;103(47):17973-8*

The two data sets correspond to gene expression measurements in human and chimpanzee brains. The unprocessed data came originally from Khaitovich et al (2004). To facilitate comparison with the original analysis, we use the microarray normalization procedures and gene selection procedures described in Oldham et al (2006).

**Analysis of microarray data**

The following description is taken from Oldham et al 2006. The dataset used for network construction consisted of 36 Affymetrix HGU95Av2 microarrays surveying gene expression with 12,625 probe sets in three adult humans and three adult chimpanzees across six matched brain regions: Broca's area, anterior cingulate cortex, primary visual cortex, prefrontal cortex, caudate nucleus, and cerebellar vermis. Because the arrays used in this study were designed for human mRNA sequences, it is necessary to account for the effect of interspecies sequence differences on chimpanzee gene-expression values. All probes on the array were compared against the human genome (Build 34) and the chimpanzee draft genome using MegaBlast (http://www.ncbi.nlm.nih.gov/BLAST/). Any probe without a perfect match in both species (approximately 1/4) was masked during the calculation of expression values (GCOSv1.2, Affymetrix, Inc). In addition, only probe sets with six or more matching probes were retained for subsequent analyses (n=11,768/12,625). For each array, expression values were scaled to an average intensity of 200 (GCOSv1.2, Affymetrix, Inc.). Quantile normalization was then performed and inter-array correlations were calculated using R. Following quantile normalization, the average inter-array correlation was 0.924 among all 18 human arrays and 0.937 among all 18 chimpanzee arrays. In specific instances in which the composition of this dataset was altered, the removal of a brain region(s) took place prior to quantile normalization.

For computational reasons, network analysis was limited to 4000 probe sets. (Note: although some genes are represented by multiple probe sets and other probe sets are not fully annotated, for consistency we refer to probe sets as "genes" throughout the manuscript, unless otherwise noted.) In order to enrich this subset with genes likely to play important roles in the brain, a non-neural "filter" was applied to identify genes with greater variance in neural versus non-neural tissue. A publicly available microarray dataset was obtained for human lung (3). The human lung dataset consisted of 18 Affymetrix HGU95Av2 microarrays measuring gene expression in normal adult human lung tissue, and was normalized as described above. The same probe mask file that was used for the human and chimpanzee brain datasets was also applied to the human lung dataset. After scaling all arrays to the same average intensity (200), the mean inter-array correlation for one human lung array (CL2001032718AA) was 2.91 SD below the average and was removed from the dataset. Following quantile normalization in R, the average inter-array correlation for the human lung dataset was 0.925. For each probe set on the microarray, the variance was calculated in the non-neural (lung) and neural (brain) datasets for the human samples. All probe sets were ranked according to their variance in both the neural and non-neural datasets, and each probe set's rank in the neural dataset was subtracted from its rank in the non-neural dataset. These rank differences (sorted from high to low) were used to select the top 4000 probe sets among all probe sets showing greater variance in human brain than human lung. In cases where <4000 probe sets showed greater variance in brain than lung, the top 4000 probe sets were selected solely on the basis of their rank differences between the neural and non-neural datasets.

**Weighted gene co-expression network construction.**

In co-expression networks, nodes correspond to genes, and connection strengths are determined by the pairwise correlations between expression profiles. In contrast to unweighted networks, weighted networks use soft thresholding of the Pearson correlation matrix for determining the connection strengths between two genes. Soft thresholding of the Pearson correlation preserves the continuous nature of the gene co-expression information, and leads to results that are highly robust with respect to the weighted network construction method (Zhang and Horvath 2005). The theory of the network construction algorithm is described in detail elsewhere (Zhang and Horvath, 2005). Briefly, a gene co-expression similarity measure (absolute value of the Pearson product moment correlation) is used to relate every pairwise gene–gene relationship. An adjacency matrix is then constructed using a “soft” power adjacency function a_ij_ = |cor(x_i_, x_j_)|^β^ where the absolute value of the Pearson correlation measures gene is the co-expression similarity, and a_ij_ represents the resulting adjacency that measures the connection strengths. The network connectivity (k_all_) of the i-th gene is the sum of the connection strengths with the other genes. The network satisfies scale-free topology if the connectivity distribution of the nodes follows an inverse power law, (frequency of connectivity p(k) follows an approximate inverse power law in k, i.e., p(k) ~ k^{−γ). Zhang and Horvath (2005) proposed a scale-free topology criterion for choosing β, which was applied here. In order to make meaningful comparisons across datasets, a power of β=9 was chosen for all analyses. This scale free topology criterion uses the fact that gene co-expression networks have been found to satisfy approximate scale-free topology. Since we are using a weighted network as opposed to an unweighted network, the biological findings are highly robust with respect to the choice of this power. Many co-expression networks satisfy the scale-free property only approximately.

**Topological Overlap and Module Detection**

A major goal of network analysis is to identify groups, or "modules", of densely interconnected genes. Such groups are often identified by searching for genes with similar patterns of connection strengths to other genes, or high "topological overlap". It is important to recognize that correlation and topological overlap are very different ways of describing the relationship between a pair of genes: while correlation considers each pair of genes in isolation, topological overlap considers each pair of genes in relation to all other genes in the network. More specifically, genes are said to have high topological overlap if they are both strongly connected to the same group of genes in the network (i.e. they share the same "neighborhood"). Topological overlap thus serves as a crucial filter to exclude spurious or isolated connections during network construction (Yip and Horvath 2007). To calculate the topological overlap for a pair of genes, their connection strengths with all other genes in the network are compared. By calculating the topological overlap for all pairs of genes in the network, modules can be identified.

The advantages and disadvantages of the topological overlap measure are reviewed in Yip and Horvath (2007) and Zhang and Horvath (2005).

**Consensus module detection**

Consensus module detection is similar to the individual dataset module detection in that it uses hierarchical clustering of genes according to a measure of gene dissimilarity. We use a gene-gene consensus dissimilarity measure Dissim(i,j) as input of average linkage hierarchical clustering. We define modules as branches of the dendrogram. To cut-off branches we use a fixed height cut-off. Modules must contains a minimum number n_0_ of genes. Module detection proceeds along the following steps:

(1) perform a hierarchical clustering using the consensus dissimilarity measure;

(2) cut the clustering tree at a fixed height cut-off;

(3) each cut branch with at least n_0_ genes is considered a separate module;

(4) all other genes are considered unassigned and are colored in ``grey''.

The resulting modules will depend to some degree on the cut height and minimum size.

**Definition of the Eigengene**

Denote by X the expression data of a given module (rows are genes, columns are microarray samples). First, the gene expression data X are scaled so that each gene expression profile has mean 0 and variance 1. Next, the gene-expression data X are decomposed via singular value decomposition (X=UDV^T^) and the value of the first module eigengene, V_1_, represents the module eigengene. Specifically, V_1_ corresponds to the largest singular value. This definition is equivalent to defining the module eigengene as the first principal component of cor(t(X)), i.e. the correlation matrix of the gene expression data.

**Consensus module analysis**

Our gene selection criteria were identical to those of Oldham et al 2006, described above. Specifically, of the 4000 genes (Affymetric microarray probesets) expressed in the brain samples, we selected the ones whose scaled connectivity (that is, connectivity normalized by the connectivity of the most connected gene) is at least 0.1 in both human and chimp samples. The Pearson correlation matrices for each dataset were turned into adjacencies by raising their absolute values to power β=9; the adjacencies were used to compute the TOM similarities in each dataset. The consensus dissimilarity was computed as minimum TOM across the two data sets. We used the consensus dissimilarity as input of average-linkage hierarchical clustering. Branches of the resulting dendrogram were identified using a fixed-height cut of 0.96 and minimum module size of 25. To determine whether some of the 9 detected initial modules were too similar, we calculated their eigengenes in each dataset, and formed their correlation matrices (one for each dataset). A consensus eigengene similarity matrix was calculated as the minimum of the data set specific eigengene correlation matrices; this matrix was turned into dissimilarity by subtracting it from one and used as input of average-linkage hierarchical clustering again. In the resulting dendrogram of consensus modules, branches with merging height less than 0.25 were identified and modules on these branches were merged. Such branches correspond to modules whose eigengenes have a correlation of 0.75 or higher, which we judge to be close enough to be merged. This module-merging procedure resulted in 7 final consensus modules that are described in our main text.

**R Software Tutorial**

A self-contained R software tutorial that illustrates how to carry out an eigengene network analysis across two datasets, together with the data can be found at the webpage

<http://www.genetics.ucla.edu/labs/horvath/CoexpressionNetwork/EigengeneNetwork>

The R code allows to reproduce the Figures and tables reported in Langfelder and Horvath (2007). Some familiarity with the R software is desirable but the document is fairly self-contained.

More material on weighted network analysis can be found at

<http://www.genetics.ucla.edu/labs/horvath/CoexpressionNetwork/>

**References**

- *Oldham M, Horvath S, Geschwind D (2006) Conservation and Evolution of Gene Co-expression Networks in Human and Chimpanzee Brains. PNAS. Nov 21;103(47):17973-8*
- *Khaitovich, P., Muetzel, B., She, X., Lachmann, M., Hellmann, I., Dietzsch, J., Steigele, S., Do, H. H., Weiss, G., Enard, W., Heissig, F., Arendt, T., Nieselt-Struwe, K., Eichler, E. E. & Paabo, S. (2004) Genome Res 14, 1462-73*
- *Zhang, B. & Horvath, S. (2005) Statistical Applications in Genetics and Molecular Biology 4, Article 17.*
- *Yip A, Horvath S (2007) Gene network interconnectedness and the generalized topological overlap measure BMC Bioinformatics 2007, 8:22*
