# Supplementary material for: Eigengene networks for studying the relationships between co-expression modules
Source: BMC Syst Biol. 2007 Nov 21;1:54. doi: 10.1186/1752-0509-1-54 (PMC2267703; doi:10.1186/1752-0509-1-54)

Comparing human–chimp consensus modules to their human data set specific counterparts

Peter Langfelder and Steve Horvath*
*Corresponding author: shorvath@mednet.ucla.edu

This additional file accompanies our main paper *Eigengene networks for studying the relationships between co-expression modules*. Presented is a comparison of the human modules published by Oldham *et al* (2006) and the consensus modules detected in our analysis in the main paper. In the table below, each row corresponds to one human-specific module and each column to a consensus module. Numbers immediately following each module label denote the total number of genes in the module. The table is a contingency table, that is, each number in the table denotes the number of genes in the intersection of the modules corresponding to the row and column. Color encodes the p-value of the Fisher exact test. The color legend shows $-log_{10}(p)$; for clarity we truncate all $p < 10^{-50}$ to 10^{-50}. Strong red color indicates the most significant overlap between the corresponding human and consensus modules. Most human modules have a counterpart consensus module with which they share a significant number of genes. The exceptions are the human green and blue modules, most of whose genes are not present in the consensus modules.

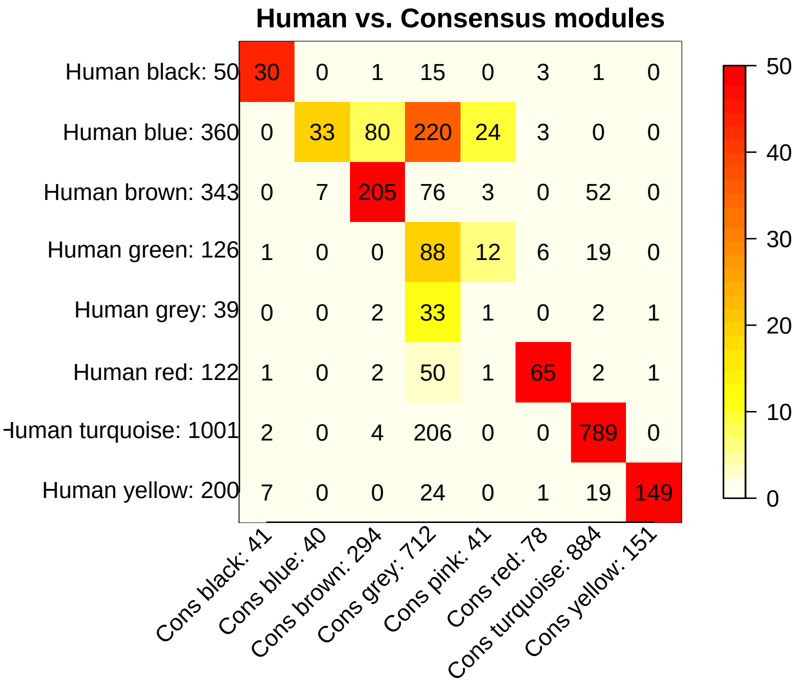

Supplement: Additional file 2 — Comparing human-chimp consensus modules to their human data set specific counterparts. This document describes a comparison between our human-chimp consensus modules and the human-specific modules detected by Oldham et al [11]. [file 1752-0509-1-54-S2.PDF]
