# Supplementary material for: Eigengene networks for studying the relationships between co-expression modules
Source: BMC Syst Biol. 2007 Nov 21;1:54. doi: 10.1186/1752-0509-1-54 (PMC2267703; doi:10.1186/1752-0509-1-54)
Supplement: Additional file 3 — Detailed description of data for Application 2, differential eigengene network analysis of expression data from four tissues in female mice. This document describes the mouse tissue microarray data sets and the module detection method. The R code posted on our web page allows one to reproduce the Figures and tables reported in the main text. [file 1752-0509-1-54-S3.DOC]

**Eigengene Network Analysis:**

**Four Tissues Of Female Mice**

Peter Langfelder and Steve Horvath

Correspondence: [shorvath@mednet.ucla.edu](mailto:shorvath@mednet.ucla.edu), [Peter.Langfelder@gmail.com](mailto:Peter.Langfelder@gmail.com)

The methods and biological implications are described in the following reference

- *Langfelder P, Horvath S (2007) Eigengene networks for studying the relationships between co-expression modules. BMC Systems Biology*

For a detailed description of the mouse intercross, see Wang et al. (2006). A description of the microarray data can be found in Ghazalpour et al (2006). To facilitate comparison with the original analysis of Ghazalpour et al (2006), we used the same gene selection in our analysis.

**Microarray Data**

The Agilent microarrays measured gene expression profiles in female mice of an F2 mouse intercross described in Ghazalpour et al 2006. The F2 mouse intercross data set (referred to as B × H cross) involved 135 female mice derived from the F2 intercross between inbred strains C3H/HeJ and C57BL/6J (Ghazalpour et al. 2006; Wang et al. 2006). B×H mice are ApoE null (ApoE −/−) and thus hyperlipidemic and were fed a high-fat diet. The B×H mice were sacrificed at 24 weeks.

**Microarray analysis**

RNA preparation and array hybridizations were performed at Rosetta Inpharmatics (Seattle, Washington, United States). The custom ink-jet microarrays used in this study (Agilent Technologies [Palo Alto, California, United States], previously described) contain 2,186 control probes and 23,574 non-control oligonucleotides extracted from mouse Unigene clusters and combined with RefSeq sequences and RIKEN full-length clones. Mouse livers were homogenized and total RNA extracted using Trizol reagent (Invitrogen, Carlsbad, California, United States) according to manufacturer's protocol. Three μg of total RNA was reverse transcribed and labeled with either Cy3 or Cy5 fluorochromes. Purified Cy3 or Cy5 complementary RNA was hybridized to at least two microarray slides with fluor reversal for 24 h in a hybridization chamber, washed, and scanned using a laser confocal scanner. Arrays were quantified on the basis of spot intensity relative to background, adjusted for experimental variation between arrays using average intensity over multiple channels, and fit to an error model to determine significance (type I error). Gene expression is reported as the ratio of the mean log10 intensity (mlratio) relative to the pool derived from 150 mice randomly selected from the F2 population.

**Microarray data reduction**

In order to minimize noise in the gene expression dataset, several data-filtering steps were taken. First, preliminary evidence showed major differences in gene expression levels between sexes among the F2 mice used, and therefore only female mice were used for network construction. The construction and comparison of the male network will be reported elsewhere. Only those mice with complete phenotype, genotype, and array data were used. This gave a final experimental sample of 135 female mice used for network construction. To reduce the computational burden and to possibly enhance the signal in our data, we used only the 8,000 most-varying female liver genes in our preliminary network construction. For module detection, we limited our analysis to the 3,600 most-connected genes because our module construction method and visualization tools cannot handle larger datasets at this point. By definition, module genes are highly connected with the genes of their module (i.e., module genes tend to have relatively high connectivity). Thus, for the purpose of module detection, restricting the analysis to the most-connected genes should not lead to major information loss. Since the network nodes in our analysis correspond to genes as opposed to probesets, we eliminated multiple probes with similar expression patterns for the same gene. Specifically, the 3,600 genes were examined, and where appropriate, gene isoforms and genes containing duplicate probes were excluded by using only those with the highest expression among the redundant transcripts. This final filtering step yielded a count of 3,421 genes for the experimental network construction.

**Weighted gene co-expression network construction.**

Constructing a weighted co-expression network is critical for identifying modules and for defining the intramodular connectivity. In co-expression networks, nodes correspond to genes, and connection strengths are determined by the pairwise correlations between expression profiles. In contrast to unweighted networks, weighted networks use soft thresholding of the Pearson correlation matrix for determining the connection strengths between two genes. Soft thresholding of the Pearson correlation preserves the continuous nature of the gene co-expression information, and leads to results that are highly robust with respect to the weighted network construction method (Zhang and Horvath 2005). The theory of the network construction algorithm is described in detail elsewhere (Zhang and Horvath, 2005). Briefly, a gene co-expression similarity measure (absolute value of the Pearson product moment correlation) is used to relate every pairwise gene–gene relationship. An adjacency matrix is then constructed using a “soft” power adjacency function a_ij_ = |cor(x_i_, x_j_)|^β^ where the absolute value of the Pearson correlation measures gene is the co-expression similarity, and a_ij_ represents the resulting adjacency that measures the connection strengths. The network connectivity (k_all_) of the i-th gene is the sum of the connection strengths with the other genes. This summation performed over all genes in a particular module is the intramodular connectivity (k_in_). The network satisfies scale-free topology if the connectivity distribution of the nodes follows an inverse power law, (frequency of connectivity p(k) follows an approximate inverse power law in k, i.e., p(k) ~ k^{−γ). Zhang and Horvath (2005) proposed a scale-free topology criterion for choosing β, which was applied here. In order to make meaningful comparisons across datasets, a power of β=6 was chosen for all analyses. This scale free topology criterion uses the fact that gene co-expression networks have been found to satisfy approximate scale-free topology. Since we are using a weighted network as opposed to an unweighted network, the biological findings are highly robust with respect to the choice of this power. Many co-expression networks satisfy the scale-free property only approximately.

**Topological Overlap and Module Detection**

A major goal of network analysis is to identify groups, or "modules", of densely interconnected genes. Such groups are often identified by searching for genes with similar patterns of connection strengths to other genes, or high "topological overlap". It is important to recognize that correlation and topological overlap are very different ways of describing the relationship between a pair of genes: while correlation considers each pair of genes in isolation, topological overlap considers each pair of genes in relation to all other genes in the network. More specifically, genes are said to have high topological overlap if they are both strongly connected to the same group of genes in the network (i.e. they share the same "neighborhood"). Topological overlap thus serves as a crucial filter to exclude spurious or isolated connections during network construction (Yip and Horvath 2007). To calculate the topological overlap for a pair of genes, their connection strengths with all other genes in the network are compared. By calculating the topological overlap for all pairs of genes in the network, modules can be identified. The advantages and disadvantages of the topological overlap measure are reviewed in Yip and Horvath (2007) and Zhang and Horvath (2005).

**Definition of the Eigengene**

Denote by X the expression data of a given module (rows are genes, columns are microarray samples). First, the gene expression data X are scaled so that each gene expression profile has mean 0 and variance 1. Next, the gene-expression data X are decomposed via singular value decomposition (X=UDV^T^) and the value of the first module eigengene, V_1_, represents the module eigengene. Specifically, V_1_ corresponds to the largest singular value.

**Consensus module analysis**

For this analysis, we started with the 3421 genes (probesets) used in Ghazalpour et al 2006. The genes were the most connected genes among the 8000 most varying genes of the female liver dataset. We filtered out 6 genes that had zero variance in at least one of the four tissue datasets, leaving 3415 genes. For each of the data sets (corresponding to the 4 tissues), the Pearson correlation matrix of the genes was calculated and turned into adjacencies by raising the absolute value to power β=6. From the adjacency matrices, we calculated the TOM similarities which were then used to calculate the consensus dissimilarity. The dissimilarity was used as input in average-linkage hierarchical clustering. Branches of the resulting dendrogram were identified using the Dynamic Tree Cut algorithm (Langfelder et al. 2007). The maximum merging height for the cutting was set to 0.97, and minimum module size to 25. This procedure resulted in 12 initial consensus modules. To determine whether some of the initial consensus modules should be merged, we calculated their eigengenes in each dataset, and formed their correlation matrices (one for each dataset). A ``minimum consensus similarity'' matrix was calculated as the minimum of the dataset eigengene correlation matrices; this matrix was turned into dissimilarity by subtracting it from one and used as input of average-linkage hierarchical clustering again. In the resulting dendrogram of consensus modules, branches with merging height less than 0.25 were identified and modules on these branches were merged. Such branches correspond to modules whose eigengenes have a correlation of 0.75 or higher, which we judge to be close enough to be merged. This module-merging procedure resulted in 11 final consensus modules that are described in the main text.

**R Software Tutorial**

A self-contained R software tutorial that illustrates how to carry out an eigengene network analysis across two datasets, together with the data can be found at the webpage

<http://www.genetics.ucla.edu/labs/horvath/CoexpressionNetwork/EigengeneNetwork>

The R code allows to reproduce the Figures and tables reported in Langfelder and Horvath (2007). Some familiarity with the R software is desirable but the document is fairly self-contained.

More material on weighted network analysis can be found at

<http://www.genetics.ucla.edu/labs/horvath/CoexpressionNetwork/>

**References**

The microarray data and processing steps are described in

- *Ghazalpour A, Doss S, Zhang B, Wang S, Plaisier C, Castellanos R, Brozell A, Schadt EE, Drake TA, Lusis AJ, Horvath S (2006) "Integrating Genetic and Network Analysis to Characterize Genes Related to Mouse Weight". PLoS Genetics. Volume 2 | Issue 8 | AUGUST 2006*

The mouse cross is described in

- *Wang S, Yehya N, Schadt EE, Wang H, Drake TA, et al. (2006) Genetic and genomic analysis of a fat mass trait with complex inheritance reveals marked sex specificity. PLoS Genet 2:e15*

Weighted gene co-expression network analysis is described in

- *Bin Zhang and Steve Horvath (2005) A General Framework for Weighted Gene Co-Expression Network Analysis, Statistical Applications in Genetics and Molecular Biology, Vol. 4: No. 1, Article 17.*

The Dynamic Tree Cut algorithm is described in

- *Peter Langfelder, Bin Zhang and Steve Horvath (2007) Defining clusters from a hierarchical cluster tree: the Dynamic Tree Cut package for R, Bioinformatics.*

Other references

- *Yip A, Horvath S (2007) Gene network interconnectedness and the generalized topological overlap measure BMC Bioinformatics 2007, 8:22*
