# Supplementary material for: Eigengene networks for studying the relationships between co-expression modules
Source: BMC Syst Biol. 2007 Nov 21;1:54. doi: 10.1186/1752-0509-1-54 (PMC2267703; doi:10.1186/1752-0509-1-54)
Supplement: Additional file 4 — Functional enrichment analysis of consensus modules across four tissues of female mice. In this document we report functional enrichment p-values provided by the online tool DAVID [20]. The results show that our consensus modules are highly significantly enriched with known gene ontologies. [file 1752-0509-1-54-S4.PDF]

Functional enrichment analysis of consensus modules across four tissues in female mice

Peter Langfelder and Steve Horvath*
*Corresponding author: shorvath@mednet.ucla.edu

This document accompanies our main article *Eigengene networks for studying the relationships between co-expression modules*. We present a table detailing the results of functional enrichment analysis of consensus modules accross four female mice tissues. The table lists selected most significant terms returned by the functional enrichment analysis performed by the online tool DAVID. The columns report the following information: Color lists the module color label as described in the main text; Count lists the percentage of genes in each Gene Category that are present in the corresponding module; and the last three columns list the unadjusted p-value as well as the Bonferroni corrections based on the number of all terms returned (Bonf All), and on the number of terms returned in each system of categories separately (Bonf Sys).

Color	System	Gene Category	Count %	P-value	Bonf All	Bonf Sys
black	SP_PIR_KW	ribonucleoprotein	30.77%	1.65e-11	8.07e-10	1.15e-10
black	GO_CC_ALL	ribonucleoprotein complex	34.62%	3.52e-09	1.72e-07	4.57e-08
black	SP_PIR_KW	ribosomal protein	23.08%	4.98e-08	2.44e-06	3.48e-07
black	KEGG_PW	MMU03010:RIBOSOME	23.08%	1.94e-07	9.5e-06	1.94e-07
black	GO_MF_ALL	structural constituent of ribosome	23.08%	4.9e-07	2.4e-05	1.96e-06
black	GO_CC_ALL	ribosome	23.08%	1.37e-06	6.7e-05	1.78e-05
black	GO_CC_ALL	non-membrane-bound organelle	34.62%	3.3e-05	0.00162	0.000429
black	GO_CC_ALL	intracellular non-membrane-bound organelle	34.62%	3.3e-05	0.00162	0.000429
black	GO_BP_ALL	protein biosynthesis	23.08%	8.13e-05	0.00398	0.00163
black	GO_BP_ALL	macromolecule biosynthesis	23.08%	0.000143	0.007	0.00286
black	GO_MF_ALL	structural molecule activity	23.08%	0.000148	0.00725	0.000592
black	GO_BP_ALL	macromolecule metabolism	42.31%	0.000188	0.00919	0.00375
blue	GO_BP_ALL	immune response	26.21%	8.79e-21	2.94e-18	1.47e-18
blue	GO_BP_ALL	defense response	28.28%	3.16e-20	1.06e-17	5.28e-18
blue	GO_BP_ALL	response to biotic stimulus	28.28%	8.83e-20	2.95e-17	1.48e-17
blue	SP_PIR_KW	membrane	37.24%	1.31e-15	4.36e-13	4.7e-14
blue	SP_PIR_KW	glycoprotein	33.10%	2.52e-14	8.42e-12	9.07e-13
blue	SP_PIR_KW	transmembrane	33.79%	4.53e-12	1.51e-09	1.63e-10
blue	GO_BP_ALL	positive regulation of immune response	8.28%	6.81e-12	2.27e-09	1.14e-09
blue	SP_PIR_KW	signal	28.28%	7.19e-12	2.4e-09	2.59e-10
blue	GO_BP_ALL	inflammatory response	9.66%	3.64e-11	1.22e-08	6.09e-09
blue	SP_PIR_KW	immune response	8.28%	4.26e-11	1.42e-08	1.53e-09
blue	GO_BP_ALL	positive regulation of organismal physiological process	8.28%	4.87e-11	1.63e-08	8.14e-09
blue	GO_BP_ALL	antigen presentation, exogenous antigen	6.21%	7.35e-11	2.45e-08	1.23e-08

blue	GO_BP_ALL	antigen presentation, exogenous antigen via MHC class II	4.83%	2.11e-10	7.05e-08	3.53e-08
blue	GO_CC_ALL	external side of plasma membrane	7.59%	8.26e-10	2.76e-07	1.32e-08
blue	GO_BP_ALL	regulation of immune response	8.28%	1.03e-09	3.44e-07	1.72e-07
blue	GO_BP_ALL	response to other organism	15.17%	1.74e-09	5.82e-07	2.91e-07
blue	GO_BP_ALL	response to pest, pathogen or parasite	14.48%	5.84e-09	1.95e-06	9.75e-07
blue	GO_CC_ALL	cell surface	8.28%	1.01e-08	3.39e-06	1.62e-07
blue	GO_BP_ALL	response to external stimulus	13.10%	1.96e-08	6.53e-06	3.27e-06
blue	GO_BP_ALL	antigen presentation	6.90%	3.07e-08	1.03e-05	5.13e-06
blue	SP_PIR_KW	immunoglobulin domain	11.72%	5.44e-08	1.82e-05	1.96e-06
blue	GO_BP_ALL	positive regulation of cellular physiological process	12.41%	5.48e-08	1.83e-05	9.16e-06
blue	GO_BP_ALL	cell death	12.41%	8.25e-08	2.75e-05	1.38e-05
blue	GO_BP_ALL	death	12.41%	9.93e-08	3.32e-05	1.66e-05
blue	GO_BP_ALL	regulation of organismal physiological process	8.28%	1.06e-07	3.53e-05	1.77e-05
blue	GO_BP_ALL	positive regulation of physiological process	12.41%	1.1e-07	3.68e-05	1.84e-05
blue	GO_BP_ALL	immune cell activation	7.59%	1.75e-07	5.85e-05	2.92e-05
blue	GO_BP_ALL	cell activation	7.59%	1.85e-07	6.18e-05	3.09e-05
blue	GO_BP_ALL	positive regulation of cellular process	12.41%	1.88e-07	6.29e-05	3.14e-05
blue	GO_BP_ALL	regulation of apoptosis	9.66%	2.35e-07	7.84e-05	3.92e-05
blue	GO_BP_ALL	regulation of programmed cell death	9.66%	2.89e-07	9.66e-05	4.83e-05
blue	GO_BP_ALL	positive regulation of apoptosis	6.90%	4.99e-07	0.000167	8.33e-05
blue	GO_MF_ALL	signal transducer activity	33.10%	5e-07	0.000167	1.25e-05
blue	GO_BP_ALL	positive regulation of programmed cell death	6.90%	5.93e-07	0.000198	9.9e-05
blue	GO_BP_ALL	antigen processing, exogenous antigen via MHC class II	4.14%	6.85e-07	0.000229	0.000114
blue	GO_BP_ALL	cytokine production	5.52%	8.24e-07	0.000275	0.000138
blue	GO_BP_ALL	induction of programmed cell death	6.21%	9.98e-07	0.000333	0.000167
blue	GO_BP_ALL	induction of apoptosis	6.21%	9.98e-07	0.000333	0.000167
blue	GO_BP_ALL	response to wounding	10.34%	1.01e-06	0.000338	0.000169
blue	GO_BP_ALL	regulation of lymphocyte differentiation	4.14%	1.01e-06	0.000338	0.000169
blue	GO_BP_ALL	apoptosis	11.03%	1.02e-06	0.000342	0.000171
blue	GO_BP_ALL	positive regulation of lymphocyte activation	4.83%	1.04e-06	0.000347	0.000173
blue	GO_BP_ALL	regulation of cytokine biosynthesis	4.83%	1.04e-06	0.000347	0.000173
blue	GO_BP_ALL	positive regulation of cell activation	4.83%	1.17e-06	0.00039	0.000195
blue	GO_BP_ALL	programmed cell death	11.03%	1.31e-06	0.000437	0.000219
blue	GO_BP_ALL	regulation of cytokine production	4.83%	1.64e-06	0.000546	0.000273

blue	GO_BP_ALL	response to stimulus	30.34%	1.83e-06	0.00061	0.000305
blue	INTP_N	IPR007110:Immunoglobulin-like	13.79%	1.95e-06	0.00065	6.81e-05
blue	GO_BP_ALL	positive regulation of biological process	12.41%	2.14e-06	0.000714	0.000357
blue	GO_BP_ALL	response to stress	15.86%	2.35e-06	0.000785	0.000393
blue	GO_BP_ALL	positive regulation of cytokine biosynthesis	4.14%	3.23e-06	0.00108	0.000539
blue	GO_BP_ALL	T cell differentiation	4.14%	3.23e-06	0.00108	0.000539
blue	GO_BP_ALL	cytokine biosynthesis	4.83%	3.34e-06	0.00112	0.000558
blue	GO_BP_ALL	cytokine metabolism	4.83%	3.67e-06	0.00123	0.000614
blue	GO_BP_ALL	positive regulation of cytokine production	4.14%	3.73e-06	0.00124	0.000622
blue	SP_PIR_KW	Immunoglobulin C region	3.45%	4.85e-06	0.00162	0.000174
blue	GO_BP_ALL	taxis	5.52%	5.06e-06	0.00169	0.000845
blue	GO_BP_ALL	chemotaxis	5.52%	5.06e-06	0.00169	0.000845
blue	GO_BP_ALL	positive regulation of protein biosynthesis	4.14%	5.6e-06	0.00187	0.000936
blue	SP_PIR_KW	phosphorylation	17.24%	6.2e-06	0.00207	0.000223
blue	GO_BP_ALL	positive regulation of T cell activation	4.14%	6.37e-06	0.00213	0.00106
blue	GO_BP_ALL	positive regulation of lymphocyte differentiation	3.45%	6.56e-06	0.00219	0.00109
blue	GO_BP_ALL	lymphocyte activation	6.21%	6.87e-06	0.00229	0.00115
blue	GO_BP_ALL	positive regulation of cellular biosynthesis	4.14%	7.22e-06	0.00241	0.0012
blue	GO_BP_ALL	lymphocyte differentiation	4.83%	8.05e-06	0.00269	0.00134
blue	GO_BP_ALL	antigen presentation, exogenous peptide antigen	2.76%	9.74e-06	0.00325	0.00163
blue	GO_BP_ALL	regulation of T cell differentiation	3.45%	1e-05	0.00335	0.00167
blue	GO_BP_ALL	regulation of lymphocyte activation	4.83%	1.1e-05	0.00368	0.00184
blue	GO_BP_ALL	regulation of protein biosynthesis	5.52%	1.18e-05	0.00395	0.00198
blue	SP_PIR_KW	mhc ii	3.45%	1.19e-05	0.00396	0.000427
blue	GO_BP_ALL	regulation of cell activation	4.83%	1.19e-05	0.00397	0.00199
blue	GO_BP_ALL	positive regulation of biosynthesis	4.14%	1.29e-05	0.00429	0.00215
blue	GO_BP_ALL	organismal physiological process	26.21%	1.3e-05	0.00434	0.00217
blue	GO_BP_ALL	tumor necrosis factor-alpha biosynthesis	2.76%	1.55e-05	0.00518	0.00259
blue	GO_BP_ALL	regulation of tumor necrosis factor-alpha biosynthesis	2.76%	1.55e-05	0.00518	0.00259
blue	GO_BP_ALL	positive regulation of tumor necrosis factor-alpha biosynthesis	2.76%	1.55e-05	0.00518	0.00259
blue	GO_MF_ALL	MHC class II receptor activity	3.45%	1.61e-05	0.00538	0.000403
blue	GO_BP_ALL	regulation of cellular biosynthesis	5.52%	1.65e-05	0.0055	0.00275
blue	GO_CC_ALL	plasma membrane	20.69%	1.78e-05	0.00595	0.000285

blue	U_S_F	transmembrane region	32.41%	1.86e-05	0.00621	0.000502
blue	PIR_SF_NM	SF001991:class II histocompatibility antigen	3.45%	1.88e-05	0.00627	9.39e-05
blue	KEGG_PW	MMU04940:TYPE I DIABETES MELLITUS	5.52%	2.14e-05	0.00714	0.000214
blue	SP_PIR_KW	inflammatory response	4.14%	2.33e-05	0.00779	0.00084
blue	KEGG_PW	MMU04650:NATURAL KILLER CELL MEDIATED CYTOTOXICITY	6.90%	2.4e-05	0.008	0.00024
blue	GO_BP_ALL	positive regulation of cell differentiation	4.14%	2.59e-05	0.00866	0.00433
blue	GO_BP_ALL	regulation of biosynthesis	5.52%	2.61e-05	0.00873	0.00437
brown	SP_PIR_KW	nucleotide-binding	15.04%	3.39e-05	0.0038	0.000746
brown	GO_MF_ALL	translation regulator activity	6.19%	4.13e-05	0.00462	0.00107
brown	INTP_N	IPR000608:Ubiquitin-conjugating enzyme, E2	4.42%	6.91e-05	0.00774	0.00076
brown	GO_CC_ALL	intracellular	52.21%	6.93e-05	0.00776	0.000762
brown	GO_BP_ALL	cellular physiological process	60.18%	7.7e-05	0.00862	0.00231
brown	SM_NM	SM00212:UBCc	4.42%	8.67e-05	0.00971	0.000607
green	SP_PIR_KW	alternative splicing	24.14%	7.5e-06	0.000375	8.25e-05
green	GO_CC_ALL	intracellular organelle	46.55%	8.88e-05	0.00444	0.000622
green	GO_CC_ALL	organelle	46.55%	9.16e-05	0.00458	0.000642
greenyellow	GO_BP_ALL	protein transport	29.17%	6.35e-05	0.00292	0.00133
greenyellow	GO_BP_ALL	establishment of protein localization	29.17%	7.81e-05	0.00359	0.00164
greenyellow	GO_BP_ALL	intracellular transport	29.17%	8.15e-05	0.00375	0.00171
greenyellow	GO_BP_ALL	establishment of cellular localization	29.17%	8.43e-05	0.00388	0.00177
greenyellow	GO_BP_ALL	cellular localization	29.17%	8.86e-05	0.00408	0.00186
greenyellow	GO_BP_ALL	protein localization	29.17%	1e-04	0.00461	0.0021
greenyellow	GO_BP_ALL	intracellular protein transport	25.00%	0.000122	0.0056	0.00255
magenta	GO_BP_ALL	immune response	38.89%	6.23e-09	1.05e-06	6.36e-07
magenta	GO_BP_ALL	defense response	41.67%	9.4e-09	1.59e-06	9.59e-07
magenta	GO_BP_ALL	response to biotic stimulus	41.67%	1.36e-08	2.29e-06	1.38e-06
magenta	SP_PIR_KW	glycoprotein	44.44%	7.55e-07	0.000128	1.51e-05
magenta	GO_BP_ALL	immune cell activation	19.44%	1.06e-06	0.00018	0.000108
magenta	GO_BP_ALL	cell activation	19.44%	1.1e-06	0.000186	0.000112
magenta	SP_PIR_KW	membrane	44.44%	6.09e-06	0.00103	0.000122
magenta	GO_MF_ALL	signal transducer activity	55.56%	7.36e-06	0.00124	7.36e-05
magenta	GO_BP_ALL	lymphocyte activation	16.67%	1.26e-05	0.00213	0.00129
magenta	BIOCARTA	m_IL12Pathway:IL12 and Stat4 Dependent Signaling Pathway in Th1 Development	13.89%	2.87e-05	0.00485	0.000316
magenta	GO_BP_ALL	apoptosis	22.22%	5.66e-05	0.00956	0.00577
pink	SP_PIR_KW	protein transport	19.23%	5.99e-05	0.00258	3e-04
pink	GO_BP_ALL	protein transport	23.08%	7.85e-05	0.00338	0.0011
pink	GO_BP_ALL	establishment of protein localization	23.08%	9.38e-05	0.00403	0.00131
pink	GO_BP_ALL	protein localization	23.08%	0.000116	0.00499	0.00162

red	GO_BP_ALL	cell cycle	43.64%	9.5e-22	1.41e-19	4.46e-20
red	GO_BP_ALL	mitotic cell cycle	25.45%	1.38e-15	2.04e-13	6.49e-14
red	KEGG_PW	MMU04110:CELL CYCLE	21.82%	5.61e-15	8.3e-13	5.61e-15
red	SP_PIR_KW	cell cycle	23.64%	1.38e-14	2.05e-12	3.04e-13
red	SP_PIR_KW	atp-binding	30.91%	1.62e-12	2.4e-10	3.57e-11
red	GO_BP_ALL	regulation of progression through cell cycle	25.45%	4.34e-12	6.42e-10	2.04e-10
red	GO_BP_ALL	regulation of cell cycle	25.45%	4.5e-12	6.65e-10	2.11e-10
red	GO_BP_ALL	M phase	20.00%	4.18e-11	6.18e-09	1.96e-09
red	GO_BP_ALL	mitosis	18.18%	4.18e-11	6.19e-09	1.97e-09
red	GO_BP_ALL	M phase of mitotic cell cycle	18.18%	4.49e-11	6.65e-09	2.11e-09
red	SP_PIR_KW	nucleotide-binding	30.91%	6.83e-11	1.01e-08	1.5e-09
red	SP_PIR_KW	cell division	16.36%	9.96e-11	1.47e-08	2.19e-09
red	SP_PIR_KW	cell cycle control	10.91%	7.35e-10	1.09e-07	1.62e-08
red	GO_BP_ALL	cell division	18.18%	1.02e-09	1.51e-07	4.8e-08
red	GO_MF_ALL	ATP binding	32.73%	1.71e-09	2.54e-07	6e-08
red	GO_MF_ALL	adenyl nucleotide binding	32.73%	2.8e-09	4.14e-07	9.8e-08
red	SP_PIR_KW	mitosis	12.73%	4.28e-09	6.33e-07	9.41e-08
red	U_S_F	nucleotide phosphate-binding region:ATP	21.82%	2.56e-08	3.79e-06	1.28e-07
red	GO_CC_ALL	chromosome	18.18%	2.84e-08	4.21e-06	4.55e-07
red	GO_BP_ALL	DNA replication initiation	9.09%	4.63e-08	6.85e-06	2.18e-06
red	SP_PIR_KW	dna replication	10.91%	5.2e-08	7.7e-06	1.14e-06
red	GO_MF_ALL	purine nucleotide binding	32.73%	6.6e-08	9.77e-06	2.31e-06
red	GO_CC_ALL	chromosome, pericentric region	10.91%	8e-08	1.18e-05	1.28e-06
red	SP_PIR_KW	phosphorylation	27.27%	1.17e-07	1.73e-05	2.57e-06
red	GO_BP_ALL	DNA-dependent DNA replication	10.91%	1.63e-07	2.42e-05	7.68e-06
red	U_S_F	domain:MCM	7.27%	3.74e-07	5.54e-05	1.87e-06
red	GO_MF_ALL	nucleotide binding	32.73%	4.25e-07	6.29e-05	1.49e-05
red	INTP_N	IPR001208:MCM	7.27%	5.99e-07	8.86e-05	7.19e-06
red	GO_BP_ALL	DNA replication	12.73%	1.41e-06	0.000209	6.62e-05
red	GO_CC_ALL	intracellular non-membrane-bound organelle	27.27%	3.39e-06	0.000502	5.42e-05
red	GO_CC_ALL	non-membrane-bound organelle	27.27%	3.39e-06	0.000502	5.42e-05
red	GO_CC_ALL	intracellular	61.82%	3.62e-06	0.000535	5.79e-05
red	GO_BP_ALL	DNA unwinding during replication	7.27%	5.25e-06	0.000777	0.000247
red	GO_CC_ALL	intracellular organelle	56.36%	8.12e-06	0.0012	0.00013
red	GO_CC_ALL	organelle	56.36%	8.43e-06	0.00125	0.000135
red	GO_BP_ALL	DNA metabolism	18.18%	1.06e-05	0.00157	5e-04
red	SM_NM	SM00350:MCM	7.27%	1.07e-05	0.00158	5.35e-05
red	SP_PIR_KW	DNA replication initiation	5.45%	2.65e-05	0.00393	0.000584
red	INTP_N	IPR006670:Cyclin	7.27%	5.11e-05	0.00756	0.000613
red	GO_MF_ALL	DNA-dependent ATPase activity	7.27%	5.32e-05	0.00787	0.00186
red	GO_CC_ALL	nucleus	40.00%	5.78e-05	0.00855	0.000924
red	PIR_SF_NM	SF001771:cyclin, A/B/D/E types	5.45%	5.79e-05	0.00857	5.79e-05

turquoise	GO_MF_ALL	protein binding	28.15%	0.000181	0.00632	0.00162
turquoise	GO_MF_ALL	receptor binding	8.89%	0.000268	0.00937	0.00241
yellow	GO_BP_ALL	hexose metabolism	10.00%	5.91e-06	0.000278	0.00016
yellow	GO_MF_ALL	glyceraldehyde-3-phosphate dehydrogenase activity	7.50%	6.01e-06	0.000283	5.41e-05
yellow	GO_MF_ALL	glyceraldehyde-3-phosphate dehydrogenase (phosphorylating) activity	7.50%	6.01e-06	0.000283	5.41e-05
yellow	INTP_N	IPR000173:Glyceraldehyde 3-phosphate dehydrogenase	7.50%	6.14e-06	0.000289	1.23e-05
yellow	GO_BP_ALL	monosaccharide metabolism	10.00%	6.2e-06	0.000291	0.000167
yellow	GO_BP_ALL	alcohol metabolism	10.00%	3.52e-05	0.00166	0.000951
yellow	GO_MF_ALL	NAD binding	7.50%	5.41e-05	0.00254	0.000487
yellow	GO_BP_ALL	cellular carbohydrate metabolism	10.00%	5.85e-05	0.00275	0.00158
yellow	GO_MF_ALL	oxidoreductase activity, acting on the aldehyde or oxo group of donors, NAD or NADP as acceptor	7.50%	7.46e-05	0.00351	0.000672
yellow	SP_PIR_KW	glycolysis	7.50%	7.89e-05	0.00371	0.000316
yellow	GO_BP_ALL	glycolysis	7.50%	0.000135	0.00633	0.00364
yellow	GO_MF_ALL	oxidoreductase activity, acting on the aldehyde or oxo group of donors	7.50%	0.000143	0.00674	0.00129
yellow	GO_BP_ALL	glucose catabolism	7.50%	0.000176	0.00828	0.00476
yellow	GO_BP_ALL	carbohydrate metabolism	10.00%	0.000177	0.00832	0.00478
yellow	GO_BP_ALL	monosaccharide catabolism	7.50%	0.000182	0.00854	0.00491
yellow	GO_BP_ALL	hexose catabolism	7.50%	0.000182	0.00854	0.00491
yellow	GO_BP_ALL	alcohol catabolism	7.50%	0.000187	0.0088	0.00506
