# Supplementary material for: Eigengene networks for studying the relationships between co-expression modules
Source: BMC Syst Biol. 2007 Nov 21;1:54. doi: 10.1186/1752-0509-1-54 (PMC2267703; doi:10.1186/1752-0509-1-54)
Supplement: Additional file 5 — Detailed description of data for Application 3, differential eigengene network analysis between female and male mice. In this document we describe the details of female and male mouse liver data and the differential eigengene network analysis. We analyze the relationship between consensus modules and clinical traits. The R code posted on our web page allows one to reproduce the Figures and tables reported in the main text. [file 1752-0509-1-54-S5.DOC]

**Eigengene Network Analysis:**

**Female-Male Mouse Liver Comparison**

**R Tutorial**

Peter Langfelder and Steve Horvath

Correspondence: [shorvath@mednet.ucla.edu](mailto:shorvath@mednet.ucla.edu), [Peter.Langfelder@gmail.com](mailto:Peter.Langfelder@gmail.com)

The data and biological implications are described in the following reference

- *Langfelder P, Horvath S (2007) Eigengene networks for studying the relationships between co-expression modules. BMC Systems Biology*

For a detailed description of the mouse intercross, see Wang et al. (2006). A description of the microarray data can be found in Ghazalpour et al (2006). To facilitate comparison with the original analysis of Ghazalpour et al (2006), we used the same gene selection in our analysis.

**Physiologic traits**

The original data Ghazalpour et al 2006 contain 26 traits, some of which are highly correlated. Here we consider 10 physiologic traits related to metabolic syndrome.

To select independent traits, we clustered the traits with dissimilarity given by correlation

subtracted from one. Branches of the dendrogram correspond to groups of highly related traits; we chose a height cutoff of 0.35 (that is, correlation of 0.65) for the branch detection. For each branch, we selected a representative trait by taking the trait that is closest to the branch ``eigentrait'', that is the first principal component of the trait matrix (we would like to emphasize that the selected traits are actually measured traits and are not composite measurements). This procedure resulted in 20 traits. For brevity we only report significance for traits for which there is at least one module whose (eigengene--trait) correlation p-value is 0.01 or less. This restriction leads to 6 potentially interesting traits which we used in the analysis of module significance used in the main text.

**Weighted gene co-expression network construction.**

In co-expression networks, nodes correspond to genes, and connection strengths are determined by the pairwise correlations between expression profiles. In contrast to unweighted networks, weighted networks use soft thresholding of the Pearson correlation matrix for determining the connection strengths between two genes. Soft thresholding of the Pearson correlation preserves the continuous nature of the gene co-expression information, and leads to results that are highly robust with respect to the weighted network construction method (Zhang and Horvath 2005). The theory of the network construction algorithm is described in detail elsewhere (Zhang and Horvath, 2005). Briefly, a gene co-expression similarity measure (absolute value of the Pearson product moment correlation) is used to relate every pairwise gene–gene relationship. An adjacency matrix is then constructed using a “soft” power adjacency function a_ij_ = |cor(x_i_, x_j_)|^β^ where the absolute value of the Pearson correlation measures gene is the co-expression similarity, and a_ij_ represents the resulting adjacency that measures the connection strengths. The network connectivity (k_all_) of the i-th gene is the sum of the connection strengths with the other genes. The network satisfies scale-free topology if the connectivity distribution of the nodes follows an inverse power law, (frequency of connectivity p(k) follows an approximate inverse power law in k, i.e., p(k) ~ k^{−γ). Zhang and Horvath (2005) proposed a scale-free topology criterion for choosing β, which was applied here. In order to make meaningful comparisons across datasets, a power of β=6 was chosen for all analyses. This scale free topology criterion uses the fact that gene co-expression networks have been found to satisfy approximate scale-free topology. Since we are using a weighted network as opposed to an unweighted network, the biological findings are highly robust with respect to the choice of this power. Many co-expression networks satisfy the scale-free property only approximately.

**Topological Overlap and Module Detection**

A major goal of network analysis is to identify groups, or "modules", of densely interconnected genes. Such groups are often identified by searching for genes with similar patterns of connection strengths to other genes, or high "topological overlap". It is important to recognize that correlation and topological overlap are very different ways of describing the relationship between a pair of genes: while correlation considers each pair of genes in isolation, topological overlap considers each pair of genes in relation to all other genes in the network. More specifically, genes are said to have high topological overlap if they are both strongly connected to the same group of genes in the network (i.e. they share the same "neighborhood"). Topological overlap thus serves as a crucial filter to exclude spurious or isolated connections during network construction (Yip and Horvath 2007). To calculate the topological overlap for a pair of genes, their connection strengths with all other genes in the network are compared. By calculating the topological overlap for all pairs of genes in the network, modules can be identified. The advantages and disadvantages of the topological overlap measure are reviewed in Yip and Horvath (2007) and Zhang and Horvath (2005).

**Definition of the Eigengene**

Denote by X the expression data of a given module (rows are genes, columns are microarray samples). First, the gene expression data X are scaled so that each gene expression profile has mean 0 and variance 1. Next, the gene-expression data X are decomposed via singular value decomposition (X=UDV^T^) and the value of the first module eigengene, V_1_, represents the module eigengene. Specifically, V_1_ corresponds to the largest singular value. This definition is equivalent to defining the module eigengene as the first principal component of cor(t(X)), i.e. the correlation matrix of the gene expression data.

**Consensus module analysis**

For this analysis, we used the 3421 genes (probesets) used in Ghazalpour et al 2006. The genes were the most connected genes among the 8000 most varying genes of the female liver dataset. For each of the data sets, the Pearson correlation matrix of the genes was calculated and turned into adjacencies by raising the absolute value to power β=6. From the adjacency matrices, we calculated the TOM similarities which were then used to calculate the consensus dissimilarity. The dissimilarity was used as input in average-linkage hierarchical clustering. Branches of the resulting dendrogram were identified using the Dynamic Tree Cut algorithm (Langfelder et al. 2007). The maximum merging height for the cutting was set to 0.995, and minimum module size to 40. This procedure resulted in 18 initial consensus modules. To determine whether some of the initial consensus modules were too close, we calculated their eigengenes in each dataset, and formed their correlation matrices (one for each dataset). A ``minimum consensus similarity'' matrix was calculated as the minimum of the dataset eigengene correlation matrices; this matrix was turned into dissimilarity by subtracting it from one and used as input of average-linkage hierarchical clustering again. In the resulting dendrogram of consensus modules, branches with merging height less than 0.25 were identified and modules on these branches were merged. Such branches correspond to modules whose eigengenes have a correlation of 0.75 or higher, which we judge to be close enough to be merged. This module-merging procedure resulted in 13 final consensus modules that are described in the main text.

**References**

The microarray data and processing steps are described in

- *Ghazalpour A, Doss S, Zhang B, Wang S, Plaisier C, Castellanos R, Brozell A, Schadt EE, Drake TA, Lusis AJ, Horvath S (2006) "Integrating Genetic and Network Analysis to Characterize Genes Related to Mouse Weight". PLoS Genetics. Volume 2 | Issue 8 | AUGUST 2006*

The mouse cross is described in

- *Wang S, Yehya N, Schadt EE, Wang H, Drake TA, et al. (2006) Genetic and genomic analysis of a fat mass trait with complex inheritance reveals marked sex specificity. PLoS Genet 2:e15*

Weighted gene co-expression network analysis is described in

- *Bin Zhang and Steve Horvath (2005) "A General Framework for Weighted Gene Co-Expression Network Analysis", Statistical Applications in Genetics and Molecular Biology: Vol. 4: No. 1, Article 17.*

The Dynamic Tree Cut algorithm is described in

- *Peter Langfelder, Bin Zhang and Steve Horvath (2007) Defining clusters from a hierarchical cluster tree: the Dynamic Tree Cut package for R, Bioinformatics.*

Other references

- *Yip A, Horvath S (2007) Gene network interconnectedness and the generalized topological overlap measure BMC Bioinformatics 2007, 8:22*

**R Software Tutorial**

A self-contained R software tutorial that illustrates how to carry out an eigengene network analysis across two datasets, together with the data can be found at the webpage

<http://www.genetics.ucla.edu/labs/horvath/CoexpressionNetwork/EigengeneNetwork>

The R code allows to reproduce the Figures and tables reported in Langfelder and Horvath (2007). Some familiarity with the R software is desirable but the document is fairly self-contained.

More material on weighted network analysis can be found at

<http://www.genetics.ucla.edu/labs/horvath/CoexpressionNetwork/>
