# Supplementary material for: Eigengene networks for studying the relationships between co-expression modules
Source: BMC Syst Biol. 2007 Nov 21;1:54. doi: 10.1186/1752-0509-1-54 (PMC2267703; doi:10.1186/1752-0509-1-54)

Comparing female–male mouse liver consensus modules to their female data set specific counterparts

Peter Langfelder and Steve Horvath*
*Corresponding author: shorvath@mednet.ucla.edu

This additional file accompanies our main paper *Eigengene networks for studying the relationships between co-expression modules*. Presented is a comparison of the co-expression modules found in female liver expression data published in Ghazalpour *et al* (2006) and the consensus modules in female and male mouse livers detected in our analysis described in the main text. In the table below, each row corresponds to one female-specific module and each column to a consensus module. Numbers immediately following each module label denote the total number of genes in the module. The table is a contingency table, that is, each number in the table denotes the number of genes in the intersection of the modules corresponding to the row and column. Color encodes the p-value of the Fisher exact test for the table. The color legend shows $-log_{10}(p)$; for clarity we truncate all $p < 10^{-50}$ to 10^{-50}. Strong red color indicates the most significant overlap between the corresponding human and consensus modules. Most female-specific modules have counterpart consensus modules with which they share a significant number of genes. The exceptions are the female salmon and light-yellow modules, whose genes are not present in the consensus modules.

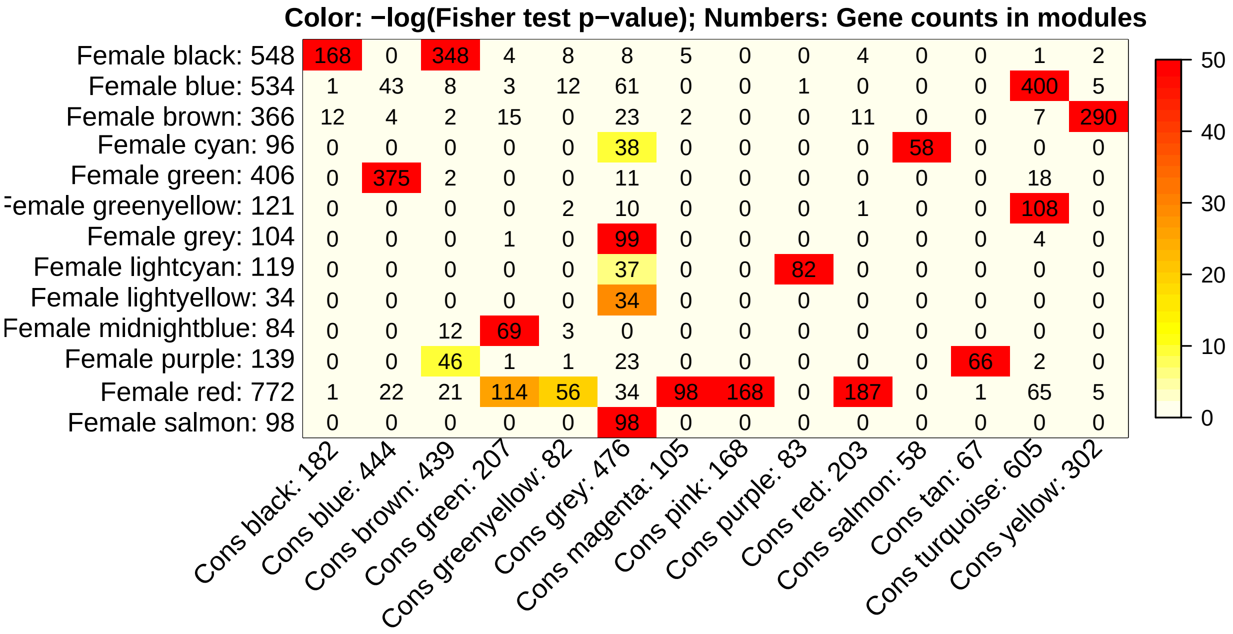

Supplement: Additional file 6 — Comparing female-male mouse liver consensus modules to their female data set specific counterparts. This document describes a comparison between our male-female mouse consensus modules and the female liver-specific modules detected by Ghazalpour et al [18]. [file 1752-0509-1-54-S6.PDF]
