# Supplementary material for: Eigengene networks for studying the relationships between co-expression modules
Source: BMC Syst Biol. 2007 Nov 21;1:54. doi: 10.1186/1752-0509-1-54 (PMC2267703; doi:10.1186/1752-0509-1-54)
Supplement: Additional file 7 — Simulation of gene expression data sets. This document describes the simulation of gene expression data sets with a modular structure. The simulation R code can be found on our web page. [file 1752-0509-1-54-S7.PDF]

Simulation of gene expression data

Peter Langfelder and Steve Horvath*
*Corresponding author: shorvath@mednet.ucla.edu

This additional file accompanies our main article *Eigengene networks for studying the relationships between co-expression modules*. Here we give a more detailed description of the simulation of gene expression data we used in the simulation study of consensus module detection. The simulation code, written in R, can be downloaded from our website http://www.genetics.ucla.edu/labs/horvath/CoexpressionNetwork/EigengeneNetwork/.

Simulating a single gene expression data set

We describe our method for simulating expression-like data sets that exhibit a modular structure similar to that observed in real gene expression data. The aim is to simulate n gene expression profiles x_i, $i = 1, .., n$, where each profile x_i is a vector with m components corresponding to m microarray samples. The l-th component of x_i will be denoted as $x_i^{(l)}$. The modular structure is specified by choosing the simulated number of modules N, module sizes $n_I, I = 1, .., N$ and a set of N module seeds (one for each module) that will be referred to as *true module eigengenes*. The module sizes are chosen such that $n > \sum_I n_I$, that is a fraction of the genes is simulated to be outside of the modules (we refer to such genes as "simulated grey"). The module seeds can be chosen in many ways, for example as independent (uncorrelated) vectors or with some correlations among them. Given that in real data eigengenes tend to exhibit non-trivial correlation patterns corresponding to meta-modules, the module seeds in our simulations were chosen to fall into a few (2–4) meta-modules.

Simulating a single module

Given a module seed $seed_I$ and desired size n_I, module genes are simulated as follows. Choose a minimum correlation r_{min} between the module genes and the seed. Generate gene expression profiles such that the correlation of the k-th module gene with the module seed is

$$\begin{aligned} \text{cor}(x_{k,I}, seed_I) &= 1 - (k/n_I)(1 - r_{min}) \quad (1) \\ &\equiv r_{k,I} \, , \quad (2) \end{aligned}$$

that is, the first gene has correlation $r_{1,I} \approx 1$ with the seed while the last (n_I-th) gene has correlation $r_{n_I,I} = r_{min}$. The required correlation (1) is achieved by calculating the k-th gene profile as the sum of the seed vector $seed_I$ and a noise term $a_k \epsilon_k$,

$$x_{k,I}^l = seed_I^{(l)} + a_k \epsilon_k^{(l)} \, , \quad (3)$$

where

$$a_k = \sqrt{\frac{\text{var}(seed_I)}{\text{var}(\epsilon_k)} \left(\frac{1}{r_{k,I}^2} - 1 \right)} . \quad (4)$$

and $\epsilon_k^{(l)} \sim N(0, 1)$. This technique produces modules consisting of genes distributed symmetrically around the module seed; in this sense, the simulated modules are spherical clusters whose centers coincide (on average) with the module seed.

Simulating genes that lie outside of the modules

Genes that are simulated to lie outside of the modules are called "simulated grey". We split such genes into two groups. The first group contains genes that are completely unrelated to any of the modules. Their expression values are chosen randomly and independently from $N(0, 1)$. The second group contains so-called "near-module" grey genes. Each of these genes lies near one of the simulated module. A near-module gene is simulated using the same procedure as the module genes, but its correlation r with the corresponding module seed is low, $r < r_{min}$ (recall that module genes are simulated with correlations between r_{min} and 1). The rationale for simulating near-module grey genes is to make the distribution of correlations around a module seed more smooth and realistic.

Submodules

Coexpression networks often exhibit modular structure in which small submodules appear to be present within the larger "main" modules. To reflect this property in the simulated data, we add an additional contribution to the generated expression profiles that corresponds to such small modules. For each simulated small module, its size is drawn from an exponential distribution with a pre-determined mean (in our simulations the mean varies between 10 and 25), and the variance of the added contribution is also drawn from an exponential distribution with mean that varies between 0.2 and 0.6. The generated contributions, representing expression profiles of the small modules, are then added to the corresponding profiles generated in the simulation of the main modules according to $x_i^{(total)} = x_i^{(main\ module)} + x_i^{(small\ module)}$. Hence the higher the variance of the generated expressions for a small module, the more pronounced the small module will be within the main modules.

Scattered small modules

It is plausible that there are causes of gene expression variability that affect expression levels of groups of genes, but membership in these groups may be completely unrelated to membership in the main modules. In other words, from the point of view of the main modules membership in such small groups appears randomly scattered and we refer to the small group as scattered modules. We simulate such small groups by simulating submodules as above, but before adding the submodule expression to the main expression profiles, the genes are randomly permuted.

Simulating a modular gene expression data set

Simulation of a data set consists of simulating the main modules and grey genes, optionally adding submodules and scattered small modules, and finally adding random noise simulated by adding independent random numbers drawn from $N(0, \sigma^2)$ to all expression values. In our simulations, the standard deviation σ of the added noise ranged from 0 to 0.3.

Simulation studies of consensus module detection

Using the simulation technique described above, two datasets of expression data were simulated in which some of the modules were consensus and others were only present in one of the two sets. We chose 6 noise levels, ranging from very clean to very noisy data (these evaluations are based on visually inspecting the clustering dendrograms). For each noise levels, we simulated 100 data set pairs and detected consensus modules using techniques identical to the actual data analysis.
